# Mediation of socioeconomic inequalities in preterm birth. A cohort analysis of Welsh linked data

**DOI:** 10.1111/aogs.15101

**Published:** 2025-04-16

**Authors:** Philip McHale, Daniela K. Schlüter, Hoda Abbasizanjani, Ashley Akbari, Ben Barr, David Taylor‐Robinson

**Affiliations:** ^1^ Department of Public Health, Policy and Systems, Institute of Population Health University of Liverpool Liverpool UK; ^2^ Population Data Science, Swansea University Medical School, Faculty of Medicine, Health & Life Science Swansea University Swansea UK

**Keywords:** epidemiology, mediation, pregnancy, preterm birth, socioeconomic inequalities

## Abstract

**Introduction:**

Consistent socioeconomic inequalities in preterm birth prevalence are seen internationally. Understanding the pathways to inequalities in preterm birth and the mediators that contribute to these inequalities is essential to inform policies and interventions to reduce health inequalities across the life course.

**Material and Methods:**

We conducted a causal mediation analysis using routinely collected, anonymised population‐scale, individual‐level linked data within the SAIL Databank on all singleton live births in Wales between 1 January, 2000 and 30 September, 2019. Our outcome was preterm birth, and exposure was area‐based deprivation. Mediators were smoking during pregnancy, maternal mental health, hospitalisation due to maternal physical health and obstetric conditions. We calculated inequalities in preterm birth (dichotomised as before or after 37 weeks) and estimated two measures of mediation: proportion eliminated, the percentage of the effect of deprivation on preterm birth eliminated by removing the mediators, through the Controlled Direct Effects; and proportion mediated, the percentage of the inequality removed by equalising the distribution of the mediators across socioeconomic strata. Multiple multivariate imputations by chained equations were used to deal with missing data.

**Results:**

The final sample included 609 610 live births with 6.1% preterm. Socioeconomic gradients were seen in preterm birth and exposure to mediators, with a higher occurrence in mothers residing in the most compared to the least deprived quintiles. Compared with the least deprived quintile, the odds ratio for preterm births in the most deprived quintile was 1.26 (95% confidence interval 1.22–1.31). The proportion eliminated by the removal of all mediators at the same time was 21%. The proportion mediated by maternal smoking during pregnancy was 26%, and less than 10% for other mediators.

**Discussion:**

Smoking during pregnancy is a significant mediator of preterm birth inequalities. Maternal mental and physical health during pregnancy and obstetric conditions also lie on the pathway from socioeconomic status to preterm birth but mediate the relationship to a lesser extent. Significant socioeconomic inequalities remained after the effect of mediators was removed. These findings suggest that there is a need to reduce inequalities in smoking during pregnancy and direct action on socioeconomic status during pregnancy.

## Graphical abstract

This is a mediation analysis of preterm birth inequalities us national linked routine data from Wales. Significant inequalities in preterm births remain after controlling for four mediators: smoking during pregnancy, maternal mental health, hospitalisation due to maternal physical health, and obstetric conditions. When using an approach that accounts for multiple mediators, smoking substantially mediated inequalities.

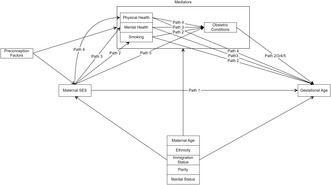

**Abbreviations**
CDE: controlled direct effect
DAG: directed acyclic graph
IDM: interventional disparity measures
PTB: preterm birth
SES: socioeconomic status
WIMD: Welsh Index of Multiple Deprivation

**Key message**

Significant inequalities in preterm births remain after controlling for four mediators: smoking during pregnancy, maternal mental health, hospitalisation due to maternal physical health, and obstetric conditions. When using an approach that accounts for multiple mediators, smoking substantially mediates inequalities.

## 1 INTRODUCTION

Preterm birth (PTB) is a major health problem, affecting 8% of live births in England and Wales in 2020.^1^ Defined as any birth that occurs before 37 weeks gestation,^2^ PTB is associated with negative consequences for respiratory and neurological development and health across the life course,^3^, ^4^ and educational outcomes.^5^

There is clear evidence that low maternal socioeconomic status (SES) is significantly associated with an increased rate of PTB.^6^ Reducing inequalities in health is an aim of both policymakers and healthcare providers, and this will be aided by identifying the key points where inequalities are generated.^7^ For example, understanding how the differential exposure and susceptibility to risk factors across socioeconomic groups will help explain the generation of health inequalities and identify areas for intervention and preventative action.

A systematic review of mediation of socioeconomic inequalities in PTB found key mediators include maternal smoking and other health behaviours, maternal physical health, maternal stress and mental health, access to antenatal healthcare, and working and environmental conditions. The review found issues in the robustness of the mediation findings due to limitations in the analytical methods (such as the underpinning assumptions and not incorporating multiple mediators).^8^ Advanced methods in mediation analysis, utilising the counterfactual approach, will allow for a more robust analysis to inform how health inequalities generate through mediation and provide evidence to support the prioritisation of intervention.^9^

This study uses data from Wales, available within the SAIL (Secure Anonymised Information Linkage) Databank,^10^ and applies advanced causal mediation methods to produce the relative contribution of mediators to inequalities in PTB. The mediators to be examined in this study are maternal smoking during pregnancy, maternal physical health, maternal mental health, and obstetric conditions.

## 2 MATERIAL AND METHODS

### 2.1 Study design and population

**FIGURE 1:**
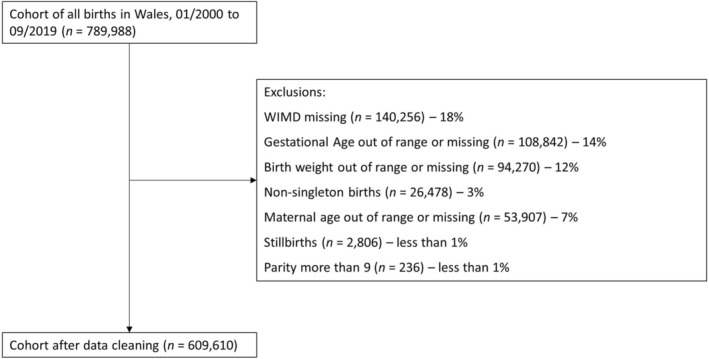
Flowchart showing construction of cohort and exclusion number. Exclusion criteria are not hierarchical or mutually exclusive, individuals may be in multiple exclusion categories. WIMD; Welsh Index of Multiple Deprivation.

We created a cohort of all births in Wales between 1 January 2000 and 30 September 2019, using anonymised, routinely collected, population‐scale, individual‐level linked data held within the SAIL Databank. We included all singleton live births with a Welsh Index of Multiple Deprivation version 2014 (WIMD, divided into quintiles) recorded, born between 22 and 43 weeks' gestation inclusive, birthweight between 500 and 6500 g, maternal age 13 to 50 inclusive, and parity of less than 10. Figure 1 shows the construction of the cohort.

### 2.2 Exposure

We measured maternal SES, our exposure of interest, using a small area‐based measure of deprivation, assessed categorically using the WIMD version 2014 quintile. WIMD is an area‐based measure of seven domains of deprivation,^11^ assigned based on the mother's Lower‐layer Super Output Area of residence, which is the smallest standard geography.^12^

### 2.3 Outcome

Our outcome of interest was PTB, dichotomised into less than 37 weeks gestation and gestation of 37 weeks or above. Additional analyses using GA at birth categorised into six groups as our outcome were conducted^2^: extremely PTB (<28 weeks), very PTB (28–31^+6^), moderate PTB (32–36^+6^), early term (37–38^+6^), term (39–41^+6^; ref), and post‐term (42 or more weeks).

### 2.4 Mediators

**FIGURE 2:**
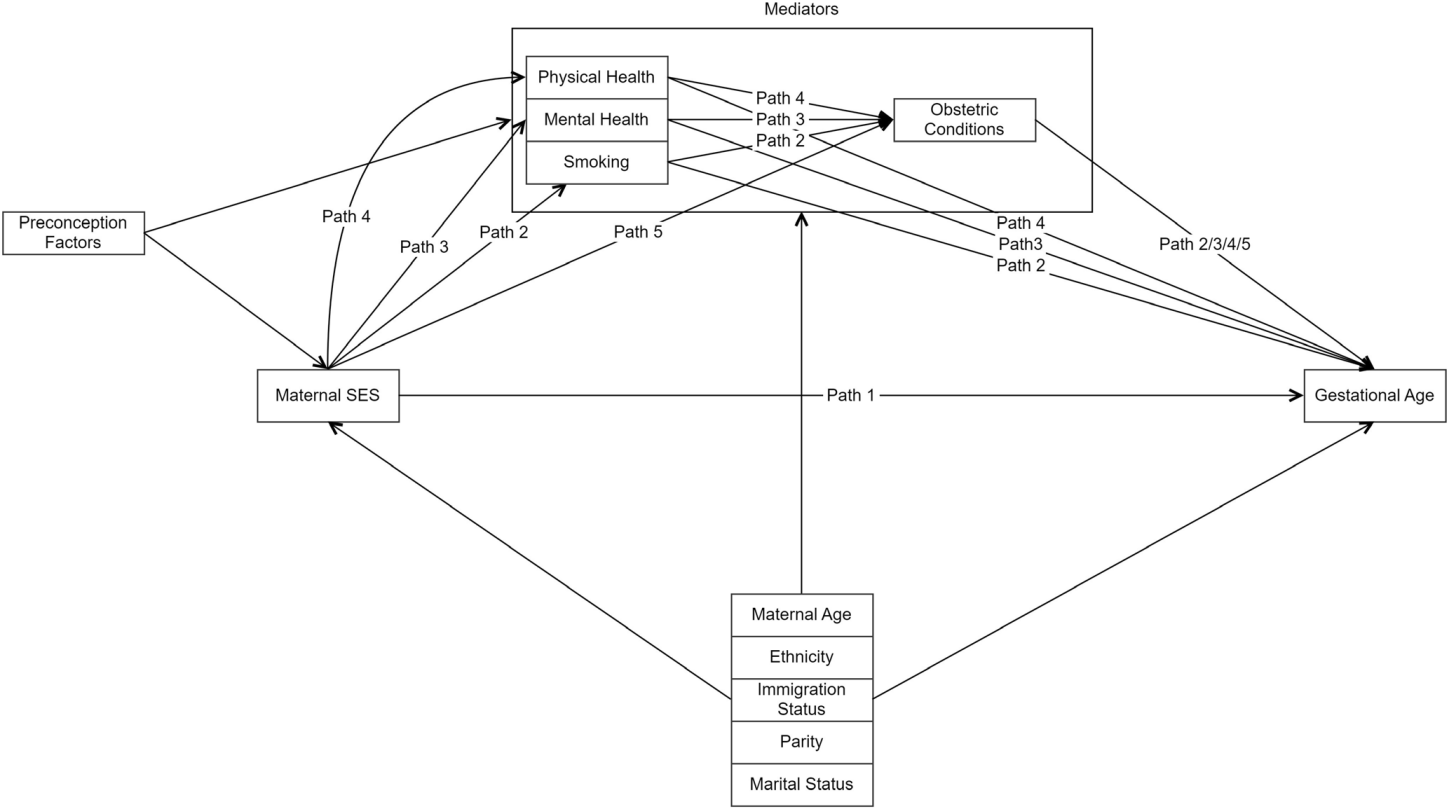
Directed acyclic graph to show the hypothesized causal pathway for mediation of preterm birth inequalities through smoking, maternal mental health, maternal physical health and maternal obstetric conditions. Path 1 = interventional disparity measure direct path; Path 2 = interventional disparity measure indirect path through smoking; Path 3 = interventional disparity measure indirect path through mental health; Path 4 = interventional disparity measure indirect path through physical health; Path 5 = interventional disparity measure indirect path through obstetric conditions; socioeconomic status (SES).

This analysis focuses on four potential mediators, represented in our directed acyclic graph (DAG; Figure 2). The four mediators are maternal smoking during pregnancy, maternal mental ill health during pregnancy, hospitalisation due to maternal physical health issues during pregnancy and obstetric conditions. The full DAG, informed by a previous systematic review, is found in c Appendix S1.^8^ Maternal mental ill health, physical health issues, and smoking are all assumed to occur independently of each other, while obstetric conditions are assumed to be dependent on the three other mediators.

Smoking status during pregnancy was identified using primary care self‐reported smoking status, recorded in the Welsh Longitudinal General Practice data source during the pregnancy period.^13^ The variable was dichotomised as current smoker or never smoked/ex‐smoker.

Maternal mental health was defined as having a recorded diagnosis with a common mental disorder flag and/or psychotic episode within the 9 months before birth, extracted using Read codes from primary care records in the Welsh Longitudinal General Practice. The combined variable was dichotomised as present or not.^14^, ^15^

Maternal physical health was defined as a dichotomous variable (hospital admission for physical health issues or not), using secondary care hospital admission records from the Patient Episode Database for Wales data source, recorded within 9 months before birth. International Classification of Diseases 10th Revision (ICD‐10) codes used for this variable are listed in Appendix S2, extracted using a published coding library,^16^ the EUROCAT guide version 1.4,^17^ and ICD‐10 chapters to inform this list. Obstetric conditions were also a dichotomised variable, using the same approach (Appendix S2).

### 2.5 Confounders

Included confounders in this analysis were parity (categorised as “none,” “one,” and “more than one”), maternal age at birth (“less than 18,” “18 to 24,” “25 to 34,” and “35 or older”)^18^ and marital status (“Within marriage/civil partnership,” “Outside marriage/civil partnership, sole registration,” “Outside marriage/civil partnership, joint registration, parents same address,” and “Outside marriage/civil partnership, joint registration, parents different address”). Other confounders in the directed acyclic graph (DAG) (ethnicity and immigration status) were not available comprehensively in our data.

### 2.6 Statistical analyses

We conducted a mediation analysis using the counterfactual method to decompose the relationship between SES and PTB into a direct path (of low SES on PTB not through mediators) and indirect paths (of SES and PTB through mediators).^9^ Our analysis estimated what would happen to inequalities in PTB if all mediators were eliminated simultaneously or if there was an intervention that acts on each mediator separately, which made the distribution equal across all WIMD quintiles.

Our DAG (Figure 2) shows the assumptions that underpin the analysis. The pathways of interest are the paths from SES to PTB (i) through smoking and from smoking via obstetric conditions (path 2), (ii) through maternal mental health and from maternal mental health via obstetric conditions (path 3), (iii) through maternal physical health and from maternal physical health via obstetric conditions (path 4), and (iv) through obstetric conditions alone (path 5). Therefore, paths 2 to 4 incorporate the indirect association through the mediator and the direct association of the mediator and obstetric conditions. Our analysis approach follows the same approach as a previous analysis.^19^

We calculated two estimates: proportion eliminated and proportion mediated. To estimate the proportion eliminated, we first calculated the controlled direct effects (CDE), which assume no unmeasured mediator‐outcome or exposure‐outcome confounding. The CDEs are a causal estimate of the effect that remains between the WIMD quintile and PTB if the mediators were removed entirely (no smoking during pregnancy, no mental health issues, or hospitalisation due to physical health or obstetric conditions).^9^ To calculate the proportion eliminated, we estimated two logistic regression models: one in which PTB is regressed on the exposure and baseline confounders (Model 1, total effect) and one where PTB is regressed on the exposure, the mediators, interaction terms between exposure and mediators, an interaction term between the mediators, and baseline confounders (Model 2). The exposure odds ratio (OR) for Model 1 is the total effect, while the CDE is the exposure OR for Model 2. The proportional difference in ORs is the proportion eliminated^9^:

$$\mathrm{PE}=\left({\text{OR}}_{\text{Model}\;1}–{\text{OR}}_{\text{Model}\;2}\right)/\left({\text{OR}}_{\text{Model}\;1}–1\right).$$

For sub‐categories of PTB, we conducted serial logistic regressions on a reduced dataset including the outcome and the reference category (term) only.

To estimate the proportion mediated, we first calculated interventional disparity measures (IDM). IDM is not a causal estimate of an exposure on an outcome; rather, it is the association between WIMD quintile and PTB if the distributions of the mediators were shifted through intervention to be the same across the population. IDMs can be used to disentangle pathways in the setting of multiple and sequential mediators.^20^ In addition to general assumptions for causal inference of consistency, no interference, and positivity, the only assumption required for identification of IDMs from observational data are that there are no unmeasured confounders of the mediator‐outcome relationship.^21^ For the case of smoking, pregnant women in WIMD quintile 1 (most deprived quintile) are simulated to smoke at the same rate as those in WIMD quintile 5 (least deprived quintile), thus removing the indirect path of SES on PTB through smoking and the effect of smoking on obstetric conditions. IDMs are presented on the absolute scale and are split into three broad measures: the Total Adjusted Association of SES on PTB, which can be split into the IDM direct path and IDM indirect path through each separate mediator.

From these measures, we calculated the proportion mediated through the indirect path of each individual mediator, with the indirect paths through other mediators incorporated in the Total Adjusted Association. A complete description of the approach calculating IDMs is included in Appendix S3. In brief, we first created richly specified logistic regression models (all 2‐way interaction terms between variables are included). We then used Monte Carlo simulation with 200 expansions (to remove error at one decimal place) to create simulated datasets. The proportion of PTB for each simulated dataset was estimated to calculate the different IDMs. All analyses were conducted in R statistical software version 4.1.3.^22^

### 2.7 Confidence intervals

Confidence intervals at 95% (95% CI) for the proportion eliminated were estimated using non‐parametric bootstrap with 1000 bootstrap samples for each of the 10 imputed datasets using the percentile method. This gave us 10 000 results, and we used the 250th and 9750th results as our confidence intervals.^23^

For proportion mediated and IDM, we used the subsampling bootstrap, resampling without replacement to a size of the original sample to the power of 0.7 and using the same approach as described above for 10 000 subsamples.^24^, ^25^ The computational requirements meant it was not feasible to calculate these in the same way as for the CDE.

### 2.8 Sensitivity analyses and robustness tests

To assess the robustness of our estimates to unmeasured confounding, we calculated E‐values for the total effect of SES on PTB and the CDE. In our study, unmeasured confounders include ethnicity and immigration status.^26^, ^27^ E‐values estimate the minimum effect size needed for an unmeasured confounder to explain the association. Reporting follows suggested best practice guidelines.^28^ As further robustness tests, proportion eliminated and proportion mediated estimates were additionally calculated using complete case analysis, and for an analysis using 34 weeks as the cutoff for PTB, imputing missing data as above.^29^ Year was considered a potential confounder (trends in mediators and outcome), so the proportion eliminated was calculated with the year included in the regression models. Marital status could be seen as a dimension of SES; therefore, we calculate the proportion eliminated when it is not included as a confounder. Finally, we calculate the proportion eliminated when smoking is split into three categories (current smoker, ex‐smoker, and never smoked).

### 2.9 Missing data

Smoking, marital status, and parity were missing in 32.9%, 1.6%, and 16.1% of cases, respectively; therefore, we used multiple multivariate imputation by chained equations. We used the R package MICE, with 10 imputations, each with 10 iterations, assuming data is missing at random.^30^ The pattern of missing data is shown in Appendix S4. We used exposure, mediators, confounders, outcomes, and year as the variables in imputation.

## 3 RESULTS

### 3.1 Sample

The final sample included 609 610 live births. Overall, 6.1% of births were preterm, 29.5% were from the most deprived WIMD quintile (6.9% preterm), and 13.7% were from the least deprived WIMD quintile (5.2% preterm). Smoking during pregnancy was present in 15.4% of births (with a clear social gradient; 22.6% in most deprived to 6.8% in least deprived). For the other mediators, 3.6% had mental health issues (4.7% in most deprived to 2.6% in least deprived), 11.2% were hospitalised due to physical health issues (12.8% in most deprived to 9.8% in least deprived), and 23.5% had obstetric conditions (25.9% in most deprived to 21.7% in least deprived). See Table 1 for full characteristics of the study population. Bar charts showing inequality in PTB and mediators are found in Appendix S5.

**TABLE 1:** Descriptive statistics of the final cleaned sample prior to imputation for missing data in smoking and parity variables (599 609 births), by exposure status, Welsh Index of Multiple Deprivation (WIMD).

	WIMD 2014 Quintile										Total	
	1 (most deprived)		2		3		4		5 (least deprived)			
	*n*	%	*n*	%	*n*	%	*n*	%	*n*	%	*n*	%
Preterm birth												
No	147 243	93.1	122 309	93.7	112 569	94.1	97 692	94.4	92 764	94.8	572 577	93.9
Yes	10 938	6.9	8254	6.3	7011	5.9	5751	5.6	5079	5.2	37 033	6.1
Smoking during pregnancy												
No	66 219	41.9	67 159	51.4	66 300	55.4	60 284	58.3	55 143	56.4	315 105	51.7
Yes	35 811	22.6	23 889	18.3	16 458	13.8	11 059	10.7	6627	6.8	93 844	15.4
Missing	56 151	35.5	39 515	30.3	36 822	30.8	32 100	31	36 073	36.9	200 661	32.9
Maternal mental health												
No	150 810	95.3	125 337	96	115 672	96.7	100 522	97.2	95 301	97.4	587 642	96.4
Yes	7371	4.7	5226	4	3908	3.3	2921	2.8	2542	2.6	21 968	3.6
Maternal physical health												
No	137 855	87.2	115 190	88.2	106 899	89.4	92 969	89.9	88 208	90.2	541 121	88.8
Yes	20 326	12.8	15 373	11.8	12 681	10.6	10 474	10.1	9635	9.8	68 489	11.2
Obstetric conditions												
No	117 287	74.1	98 968	75.8	92 236	77.1	81 393	78.7	76 623	78.3	466 507	76.5
Yes	40 894	25.9	31 595	24.2	27 344	22.9	22 050	21.3	21 220	21.7	143 103	23.5
Maternal age												
<18	5308	3.4	3233	2.5	2134	1.8	1320	1.3	803	0.8	12 798	2.1
18–24	59 601	37.7	40 092	30.7	29 888	25	20 641	20	13 428	13.7	163 650	26.8
25–34	77 039	48.7	70 317	53.9	67 654	56.6	60 671	58.7	59 252	60.6	334 933	54.9
35+	16 233	10.3	16 921	13	19 904	16.6	20 811	20.1	24 360	24.9	98 229	16.1
Parity												
0	54 902	34.7	51 552	39.5	50 473	42.2	42 267	40.9	38 584	39.4	237 778	39
1	40 230	25.4	35 072	26.9	33 698	28.2	28 525	27.6	28 927	29.6	166 452	27.3
2+	35 991	22.8	23 654	18.1	19 711	16.5	15 284	14.8	12 534	12.8	107 174	17.6
Missing	27 058	17.1	20 285	15.5	15 698	13.1	17 367	16.8	17 798	18.2	98 206	16.1
Marital status												
Married/civil partnership	47 041	29.7	48 264	37	54 354	45.5	54 007	52.2	61 209	62.6	264 875	43.4
Not married, sole registration	17 906	11.3	10 278	7.9	6734	5.6	4312	4.2	2891	3	42 121	6.9
Not married, joint registration, parents same address	59 994	37.9	51 260	39.3	44 181	36.9	35 321	34.1	26 838	27.4	217 594	35.7
Not married, joint registration, parents different address	30 954	19.6	18 885	14.5	12 196	10.2	7724	7.5	5260	5.4	75 019	12.3
Missing	2286	1.4	1876	1.4	2115	1.8	2079	2	1645	1.7	10 001	1.6

Appendix S6 shows the distribution of variables in the excluded data. PTB was more prevalent among excluded cases; however, over 5% of this was in non‐singleton pregnancies. Maternal mental health, physical health, and obstetric conditions were less prevalent among excluded cases. Prevalences were broadly similar between the excluded and the final included data for WIMD quintile, maternal age, parity, marital status and smoking during pregnancy. Appendix S7 shows the distribution of mediators and PTB by year. PTB showed no real trend over the period, while smoking fell slightly. Both mental health and hospitalisation due to physical health saw a consistent increase in prevalence over the study period.

### 3.2 Controlled direct effect

**FIGURE 3:**
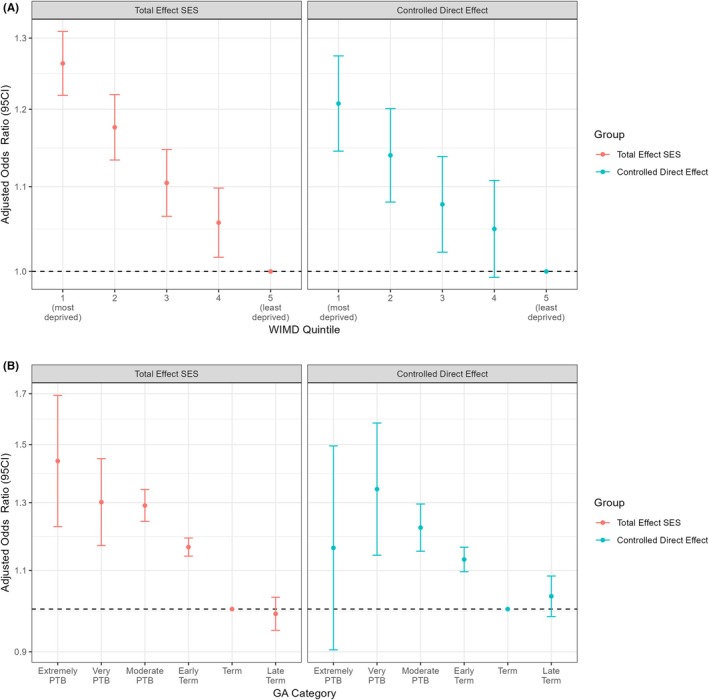
Panel A – Adjusted Odds Ratios for Welsh Index of Multiple Deprivation (WIMD) quintiles in mediator‐unadjusted (left panel) and mediator‐adjusted (controlled direct effects; right panel) models for preterm birth. Panel B – Adjusted Odds Ratios for WIMD quintile 1 compared to 5 in mediator‐unadjusted (left panel) and mediator‐adjusted (controlled direct effects; right panel) models for different gestational age categories. Mediators included are maternal smoking, mental health, physical health and obstetric conditions.

When adjusting for confounders, we estimated that the odds of PTB in mothers from the most deprived quintile were 1.26 times as high as in mothers from the least deprived quintile (95% CI 1.22, 1.31), showing a clear social gradient. The CDE for quintile 1 versus 5 is 1.21 (95 CI 1.14, 1.27) (Figure 3 panel A).

Figure 3 panel B shows the adjusted odds ratios (AOR) for each GA category, compared with term, for the most deprived WIMD quintile compared to the least deprived quintile estimated by the total effects model (left panel) and for the CDE model (right panel). All GA categories prior to term had odds significantly higher than term birth among the most deprived for the total effects models. The AOR for extremely PTB CDE was not significant (1.16, 95% CI 0.90, 1.49), but the CDEs for very PTB, moderately PTB, and early term were all significant. Full regression outputs can be found in Appendix S8.

### 3.3 Interventional disparity measures

The expected difference in the prevalence of PTB WIMD quintile 1 and WIMD quintile 5 is 1.3% points (total adjusted association). The IDM direct path is 0.7% points, while the IDM indirect path of SES through smoking was 0.3% points. Small IDM indirect paths were seen through other mediators; mental ill health was less than 0.05% (0.3% of total), hospitalisation due to physical health issues was 0.1% (1% of total), and obstetric conditions were 0.1% (2% of total). Only the IDM indirect path through smoking was significant. Full output is in Appendix S9.

### 3.4 Proportion eliminated and proportion mediated

We estimated the proportion eliminated to be 21.1% (95 CI 3.2%, 39.4%). This is the percentage of the odds ratio of PTB in mothers in the most deprived versus least deprived WIMD quintile that would be reduced if all mediators were removed. The proportion mediated through smoking was 26.0%, mental ill health 1.3%, hospitalisation due to physical health issues 6.7%, and obstetric conditions 8.5% (Table 2). This is a measure of how the association of WIMD quintile with PTB would change if, for mothers in WIMD quintile 1, the distribution of individual mediators was set to those mothers who were WIMD quintile 5.

**TABLE 2:** Proportion eliminated and proportion mediated estimates for socioeconomic inequalities in preterm birth for imputed analysis.

Proportion eliminated—of the effect of low SES (WIMD quintile 1 vs. 5) through the removal of mediators	21.1 (3.2 to 39.4)
Proportion of low SES (WIMD quintile 1 vs. 5) association Mediated by Smoking	26.0 (−101.2 to 227.5)
Proportion of low SES (WIMD quintile 1 vs. 5) association Mediated by Maternal Mental Ill Health	1.3 (−12.2 to 30.0)
Proportion of low SES (WIMD quintile 1 vs. 5) association Mediated by Maternal Physical Health Issues	6.7 (−21.1 to 55.6)
Proportion of low SES (WIMD quintile 1 vs. 5) association Mediated by Maternal Obstetric Conditions	8.5 (−41.7 to 73.0)

*Note*: Proportion eliminated is calculated through controlled direct effects, including all mediators, and proportion mediated is calculated through interventional disparity measures.

Abbreviations: SES, socioeconomic status; WIMD, Welsh Index of Multiple Deprivation.

### 3.5 Sensitivity analyses and robustness tests

Calculated E‐values were compared to AOR for measured confounders, with findings robust if no confounder AOR was larger than the lower confidence limit E‐value. Both total effects and CDE for WIMD quintile 1 were robust.

Appendix S10 shows the proportion estimates for the sensitivity analyses. For the complete case analysis, results remain broadly unchanged, with the proportion eliminated no longer significant. Proportion eliminated estimates were lower when using 34 weeks as the cut‐off (12.2%), while proportion mediated was broadly similar. The proportion eliminated was similar when the year was included. AOR for WIMD quintile 1 increased in the total effect model and proportion eliminated was 30% when marital status was excluded as a confounder. Splitting smoking into three categories produced a similar proportion eliminated, and the AOR for ex‐smokers was not significantly different from never smoked.

## 4 DISCUSSION

Using linked, routinely collected data for singleton births in Wales between 2000 and 2019, we demonstrated significant socioeconomic inequalities (assessed using WIMD) in rates of PTB that are not fully explained by mediators. If no mothers smoked, had mental ill health, were hospitalized due to physical health problems or obstetric conditions, inequalities would reduce by a fifth. Considering each mediator separately, if all mothers in the sample smoked at the same rate as mothers in the least deprived quintile, inequalities would reduce by a quarter. This variation between the proportion eliminated and the proportion mediated for smoking is likely a reflection of the confidence intervals of the two estimates. SES had small indirect paths through maternal mental health, physical health, or obstetric conditions when other mediators are considered.

Our finding that smoking is a significant mediator of SES–PTB relationship is in keeping with existing evidence,^8^ and the mediated effect is similar to that seen in the strongest evidence published to date.^31^ Our findings regarding mental and physical health are contradictory; we find neither is a significant mediator of PTB inequalities. This may be due to measurement issues in how we have defined these mediators or because other studies do not account for multiple mediators. In settings with multiple mediators, the proportion mediated estimates for each mediator, if they are assessed separately, may combine to be greater than 100%.^32^ The indirect effect through smoking may explain the effects seen through other mediators in other studies, as they do not capture indirect effects through other unmeasured mediators. Mediated effects are influenced by the prevalence of the mediator, potentially limiting the generalisability of our findings.

The proportion eliminated particularly relies on the ability to prevent the mediator from occurring. For smoking, this is relevant; however, for the other mediators, this is more complex. Preventing hospital admissions would be a feasible approach; however, preventing physical and mental health problems from occurring would require earlier intervention before pregnancy. Thus, our proportion‐eliminated estimate should be interpreted cautiously.

The Fundamental Cause Theory addresses the issue of residual inequalities and argues that unequal access to resources across social strata, including income, education, and connections, leads to an imbalance of power.^33^ This is the source of health inequities, rather than the disparities outlined in the Diderichsen model. Our results fit with both theories, suggesting that socioeconomic inequalities in PTB are explained only partly by differential exposure and susceptibility to risk factors.

Our study has several significant strengths. Using a large, nationally representative sample minimises the risk of selection bias. Additionally, we can separate the effects of multiple mediators through the analysis methods we use, and calculating IDM only assumes no unmeasured mediator‐outcome confounding, meaning unmeasured exposure‐mediator confounding (preconception factors) does not influence the robustness of our findings.

Our CDE estimates required no unmeasured mediator‐outcome or exposure‐outcome confounding. The E‐values suggest our estimates are robust. CDEs represent a different outcome from IDMs; the mediators are completely removed rather than equalised; thus, the inclusion of these estimates provides different, policy‐relevant intelligence. Our E‐values suggest our results are robust to unmeasured exposure‐outcome confounding (ethnicity and immigration status). The DAG in Appendix S1 suggests these assumptions are robust, and given that the analysis was evidence‐based, we are confident these are valid assumptions.

Despite these strengths, limitations are noted. First, other mediators, such as environmental conditions or domestic violence, are not available and may explain some of the mediated effects. This implies that other mediators, in particular, could explain the indirect pathways through smoking. However, given the consistency of smoking as the foremost mediator of PTB inequalities, it means that while this possibility cannot be discounted, the likelihood is reduced.^8^

Second, a causal interpretation of the exposure‐mediator‐outcome relationship is not possible due to unavailable data on preconception factors. Natural effects to causally explain the mediated pathways and interventional effects, which are based on a hypothesised intervention on the exposure (SES) both assume no unmeasured exposure‐mediator confounders, which are unavailable.^20^ Natural effects also assume there are no confounders of the mediator‐outcome relationship, which are affected by the exposure.^9^ We are, however, able to comment causally on the inequality change through action on the mediator.^21^ Linked with this, our assumptions of the causal ordering and independence of mediators could be challenged; however, this is based on an evidence‐based approach to construct our DAG.

The measurement of mediators may introduce bias. Smoking has been shown to be underreported among pregnant women.^34^ Our measure of physical health and obstetric conditions relies on hospital records; thus, those who are managed in primary care will not be captured. It is possible that individuals who received a primary care diagnosis in the past could either be unaffected by the condition or have already resolved it. Therefore, using only those who have a hospital admission reduces the influence of well‐managed or resolved conditions, as admissions for such conditions are unlikely to occur. Conversely, chronic conditions are unlikely to require hospitalisation in our population of pregnant women. Additionally, physical health is considered a dichotomous variable, whereas the relationship with PTB varies by specific condition.^8^ Potentially, the indirect effects through specific physical health conditions may be obscured by grouping them with conditions that do not mediate inequalities or possibly mediate in the other direction. This may also be true for mental health and obstetric conditions. The results show that general hospital admission for physical health or obstetric conditions does not mediate inequalities. Our measure of mental health includes only those being medically managed; we are unable to comment on the effect of stress or subclinical mental health problems. This may lead to an underestimation of our findings regarding indirect paths and effects.

Using area‐based measures of SES to assess inequalities only incorporates part of the inequality domain,^35^ potentially underestimating the inequality.^36^ This may explain the lower level of inequality that we found. The high number of excludes may bias the relationship between SES and PTB; however, this is unavoidable in the data we use.

Our method of estimating confidence intervals for the IDMs is a limitation. We used an approach that has been suggested in other similar settings^25^; however, we cannot discount the potential that these parameters were small, and the estimated imprecision for IDMs, and thus proportion mediated, is overly conservative. We estimated confidence intervals using subsampling and set the random number seed differently three times in total, and the same significance pattern was seen each time, giving some confidence in our imprecision estimates.

The number of imputations we use to deal with missing data are potentially too small for the confidence estimates. Due to the computational requirements, 10 imputations were used, likely robust for our point estimates; however, this may not be enough to reduce variability in our confidence estimates.^37^

## 5 CONCLUSION

Dealing directly with socioeconomic deprivation (through reducing poverty levels) locally is outside of the local policymaker's sphere of influence. Therefore, action on modifiable mediators would be a more successful intervention at a local level. Our findings show that smoking during pregnancy is a key mediator of inequalities. Smoking in pregnancy remains common in disadvantaged areas, impacting over a fifth of pregnancies in the most disadvantaged quintile of areas in Wales in our sample.

Smoking is the main mediator, which follows the mechanisms of the relationship; vasoconstriction, fetal hypoxia, and disruption of pregnancy‐related hormones are all hypothesised mechanisms for this relationship.^38^ Smoking also shows a dose–response relationship with PTB.^39^ The measure of stress used (diagnosed mental health conditions) did not mediate inequalities, which is surprising given the biological mechanism is established; the influence on the central nervous system, endocrine, immune, and vascular systems combines with genetic and epigenetic factors to lead to PTB. Additionally, stress may interact with other risk factor mediators to exacerbate the effects.^40^ This variability is potentially due to the measure used inadequately capturing stress. The remaining inequalities may represent effects through unmeasured mediators or the other hypothesised mechanisms.^41^

The data limitations are important to consider when interpreting our findings, as they may bias our results and influence the policy implications. Repeating this analysis in birth cohort studies, which include a rich set of variables, would be a beneficial accompaniment to this analysis. Importantly, richer information from different sources could be used to better identify the preventable aspects of our mediators (physical and mental health, obstetric conditions).

Smoking cessation support using financial incentives has been shown to be effective,^42^ and focussed services on deprived women or areas would potentially be helpful. The effect of interventions for smoking cessation in pregnancy on inequalities in maternal smoking is not well evidenced, and targeting of support on low socioeconomic groups is needed.^43^ A recent systematic review found that individual‐level interventions could support smokers from a low SES; however, improvements to targeted support would be needed to reduce inequalities.^44^ Further qualitative work is needed to understand how to stop smoking during pregnancy, and issues such as the targeting of support in disadvantaged communities should be considered.

There are options to influence inequalities at a service level. The Health Foundation has suggested a framework, which means the NHS adapting to better meet the social needs of patients and using resources to improve the community and working in partnership with organisations to either connect patients with appropriate partners (for example, welfare advice) or better identify and be responsive to needs.^45^ Screening for social determinants of health has also been suggested in a clinical setting and could make the NHS more responsive and better equipped to refer to appropriate support services.^46^

Another option is to “poverty‐proof” services. Initially applied to schools, this concept has been extended to healthcare settings, with barriers such as transport, appointment availability, and digital exclusion highlighted, and a need to work with stakeholders to identify need.^47^ Work is currently underway to poverty‐proof maternity services.^48^ Action on smoking, proportionate to the level of need, and local action to reduce socioeconomic inequalities are two important approaches to improving perinatal outcomes and inequalities in PTB.

## AUTHOR CONTRIBUTIONS

Philip McHale: Conceptualization, data curation, formal analysis, methodology, visualization, writing—original draft. Daniela K Schlüter: Conceptualization, formal analysis, methodology, supervision. Hoda Abbasizanjani and Ashley Akbari: Data curation, writing, review, and editing. Ben Barr and David Taylor‐Robinson: Conceptualization, supervision, writing—review & editing.

## FUNDING INFORMATION

PM is funded through an MRC Clinical Research Training Fellowship (MR/T00794X/1). DTR is supported by the NIHR Public Health Policy Research and by the NIHR on a Research Professorship (NIHR302438). BB is supported by the NIHR Applied Research Collaboration Northwest Coast (NWC ARC; Award ID: NIHR200182). BB and DTR are supported by the NIHR School for Public Health Research.

## CONFLICT OF INTEREST STATEMENT

The authors declare no conflicts of interest.

## ETHICS STATEMENT

Anonymised data were used in this study held within the SAIL Databank. Ethics approval was not required as this was a secondary analysis of anonymised data. All research conducted has been completed under the permission and approval of the SAIL independent Information Governance Review Panel (IGRP) project number 0924. Further details of this process can be found on the SAIL Databank website (https://saildatabank.com/).

## Supporting information


**Appendix S1.** Comprehensive DAG demonstrating the path from maternal SES at conception to preterm birth.
**Appendix S2.** Codes for maternal health flags.^2,3^
**Appendix S3.** Analysis plan for calculation of interventional disparity measures (IDMs).
**Appendix S4.** Pattern of missing data.
**Appendix S5.** Inequality in preterm birth and mediators.
Figure A: Distribution of preterm births in the cohort across quintiles of Welsh Index of Multiple Deprivation (IMD). The *y* axis is the percentage of total births which are preterm.
Figure B: Distribution of smoking during pregnancy in the cohort across quintiles of Welsh Index of Multiple Deprivation (IMD). The *y* axis is the percentage of total births.
Figure C: Distribution of maternal mental ill health in the cohort across quintiles of Welsh Index of Multiple Deprivation (IMD). *Y* axis is percentage of total births.
Figure D: Distribution of maternal physical health conditions in the cohort across quintiles of Welsh Index of Multiple Deprivation (IMD). *Y* axis is percentage of total births.
Figure E: Distribution of maternal obstetric conditions in the cohort across quintiles of Welsh Index of Multiple Deprivation (IMD). The *y* axis is the percentage of total births.
**Appendix S6.** Distribution of excluded data.
**Appendix S7.** Distribution of variables by year (PTB for full cohort, mediators for complete case).
**Appendix S8.** Full regression outputs for controlled direct effect (CDE) estimates.
Table A: Model results and 95% confidence intervals for total effects logistic non‐mediator adjusted regressions (exposure and confounders), for preterm birth (<37 weeks).
Table B: Model results and 95% confidence intervals for total effects logistic mediator adjusted regressions (exposure, mediators and confounders), for preterm birth (<37 weeks).
Table C: Model results and 95% confidence intervals for total effects logistic non‐mediator adjusted regressions (exposure and confounders), with each gestational age category compared to term (between 39 weeks and 41 + 6 weeks).
Table D: Model results and 95% confidence intervals for total effects logistic mediator adjusted regressions (exposure, mediators and confounders), with each gestational age category compared to term (between 39 weeks and 41 + 6 weeks).
**Appendix S9.** Interventional disparity measures.
Table E: Interventional disparity measures (IDM) as an absolute percentage change for preterm birth, as a percentage of the total, DE, direct effect; IE, indirect effect; MD, mediated dependence; TAA, total adjusted association.
**Appendix S10.** Full regression outputs for CDE estimates and IDMs for sensitivity analyses.
Table F: Model results and 95% confidence intervals for total effects logistic non‐mediator adjusted regressions (exposure and confounders), for preterm birth (<37 weeks), complete case analysis.
Table G: Model results and 95% confidence intervals for total effects logistic mediator adjusted regressions (exposure, mediators and confounders), for preterm birth (<37 weeks), complete case analysis.
Table H: Interventional disparity measures (IDM) as an absolute percentage change for preterm birth.
Table I: Model results and 95% confidence intervals for total effects logistic non‐mediator adjusted regressions (exposure and confounders), for preterm birth (<34 weeks).
Table J: Model results and 95% confidence intervals for total effects logistic mediator adjusted regressions (exposure, mediators and confounders), for preterm birth (<34 weeks), complete case analysis.
Table K: Interventional disparity measures (IDM) as an absolute percentage change for preterm birth, as a percentage of the total.
Table L: Panel A—Proportion eliminated and proportion mediated estimates for socioeconomic inequalities in preterm birth for complete case analysis. Proportion eliminated calculated through controlled direct effects, including all mediators, and proportion mediated calculated through interventional disparity measures. Panel B—Imputed analysis using 34 weeks as the cut off for preterm birth.
Table M: Model results and 95% confidence intervals for total effects logistic non‐mediator adjusted regressions (exposure and confounders), for preterm birth (<37 weeks), with year included.
TableN: Model results and 95% confidence intervals for total effects logistic mediator adjusted regressions (exposure, mediators and confounders), for preterm birth (<37 weeks) with year included.
Table O: Model results and 95% confidence intervals for total effects logistic non‐mediator adjusted regressions (exposure and confounders), for preterm birth (<37 weeks), with marital status not included.
Table P: Model results and 95% confidence intervals for total effects logistic mediator adjusted regressions (exposure, mediators and confounders), for preterm birth (<37 weeks) with marital status not included.
Table Q: Model results and 95% confidence intervals for total effects logistic mediator adjusted regressions (exposure, mediators and confounders), for preterm birth (<37 weeks) with smoking split into three categories.

## Data Availability

The data used in this study are available in the SAIL Databank at Swansea University, Swansea, UK, but as restrictions apply, they are not publicly available. All proposals to use SAIL data are subject to review by an independent Information Governance Review Panel (IGRP). Before any data can be accessed, approval must be given by the IGRP. The IGRP gives careful consideration to each project to ensure proper and appropriate use of SAIL data. When access has been granted, it is gained through a privacy‐protecting safe haven and remote access system referred to as the SAIL Gateway. SAIL has established an application process to be followed by anyone who would like to access data via SAIL at https://www.saildatabank.com/application‐process.
