# Supplementary material for: Mediation of socioeconomic inequalities in preterm birth. A cohort analysis of Welsh linked data
Source: Acta Obstet Gynecol Scand. 2025 Apr 16;104(6):1081–91. doi: 10.1111/aogs.15101 (PMC12087501; doi:10.1111/aogs.15101)
Supplement: Supplementary file 1 — Appendix S1. Comprehensive DAG demonstrating the path from maternal SES at conception to preterm birth. Appendix S2. Codes for maternal health flags.2,3 Appendix S3. Analysis plan for calculation of interventional disparity measures (IDMs). Appendix S4. Pattern of missing data. Appendix S5. Inequality in preterm birth and mediators. Figure A: Distribution of preterm births in the cohort across quintiles of Welsh Index of Multiple Deprivation (IMD). The y axis is the percentage of total births which are preterm. Figure B: Distribution of smoking during pregnancy in the cohort across quintiles of Welsh Index of Multiple Deprivation (IMD). The y axis is the percentage of total births. Figure C: Distribution of maternal mental ill health in the cohort across quintiles of Welsh Index of Multiple Deprivation (IMD). Y axis is percentage of total births. Figure D: Distribution of maternal physical health conditions in the cohort across quintiles of Welsh Index of Multiple Deprivation (IMD). Y axis is percentage of total births. Figure E: Distribution of maternal obstetric conditions in the cohort across quintiles of Welsh Index of Multiple Deprivation (IMD). The y axis is the percentage of total births. Appendix S6. Distribution of excluded data. Appendix S7. Distribution of variables by year (PTB for full cohort, mediators for complete case). Appendix S8. Full regression outputs for controlled direct effect (CDE) estimates. Table A: Model results and 95% confidence intervals for total effects logistic non‐mediator adjusted regressions (exposure and confounders), for preterm birth (<37 weeks). Table B: Model results and 95% confidence intervals for total effects logistic mediator adjusted regressions (exposure, mediators and confounders), for preterm birth (<37 weeks). Table C: Model results and 95% confidence intervals for total effects logistic non‐mediator adjusted regressions (exposure and confounders), with each gestational age category compared to term (between 39 wee [file AOGS-104-1081-s001.docx]

# Appendix S1 – Comprehensive DAG demonstrating the path from maternal SES at conception to preterm birth


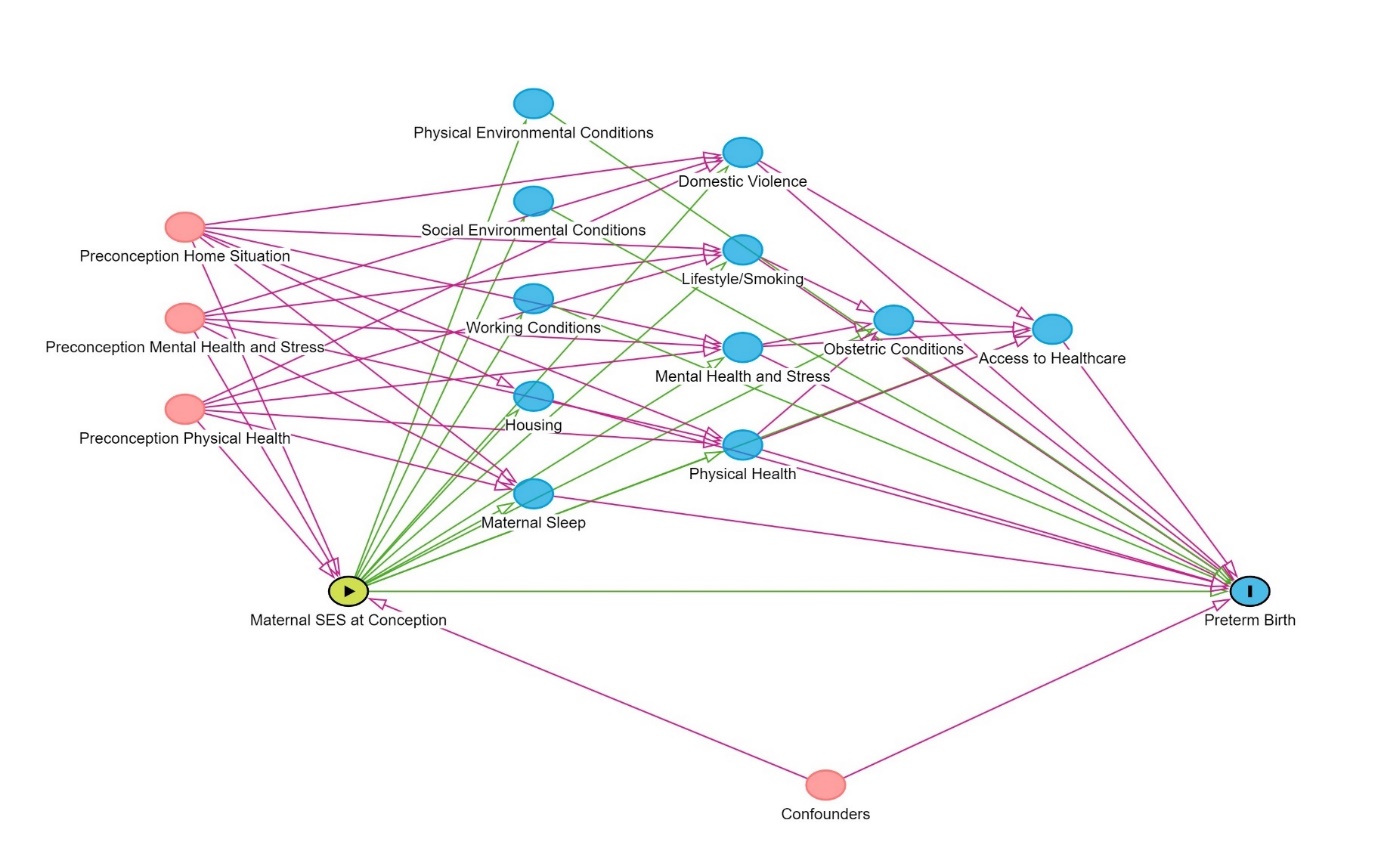


Hypothesised DAG incorporating complete collection of mediators of the pathway from Maternal Socioeconomic Status (SES) at Conception to Preterm Birth based on systematic review by McHale *et al*.^1^

# Appendix S2 – Codes for maternal health flags^2,3^

Pre-existing (9 months prior to birth)

1. Hypertension: I10 to I15
2. Diabetes: E10 to E14, O240-O243, G590, G632, H280, H360, M142, N083
3. Asthma: J45 and J46
4. Epilepsy: G40 and G41
5. Chronic alcoholism: F102
6. Drug addiction: F112, F122, F132, F142
7. Thyroid: E00 to E03, E05, E062, E063, E065, E069, H062
8. Migraine: G43
9. Eating disorders: F50
10. Obesity: E66
11. Metabolic disorders: E70 to E88
12. Cancer: C00-C97
13. Circulatory: I05-I09, I20-I89
14. Genitourinary: N00-N39, N70-N98, D25-D26
15. Joint: M45, M46, M05-M14, M30-M36
16. Gastrointestinal: K20 – K90, K92, K93
17. Haematology: D50-D89 (includes anaemia)

Obstetric Conditions (9 months prior to birth)

1. Gestational diabetes: O244 and O249
2. Gestational Hypertension (including pre-eclampsia/eclampsia): O10, O11, O13-O16
3. Gestational [pregnancy-induced] oedema and proteinuria without hypertension: O12
4. Genitourinary Infections: O23
5. Haemorrhage in early pregnancy: 020
6. Hyperemesis: O21
7. Excessive Gestational weight gain: O260
8. Malnutrition: O25
9. Venous complications and other; O22, O261-O267, O269;
10. Antepartum Haemorrhage: O46
11. Maternal care for known or suspected abnormality of pelvic organs: O34
12. Infection: based on EuroCAT
    1. Coxsackie’s B341
    2. Influenza J10 - J11
    3. Listeria A32
    4. Mumps B26
    5. Rubella B06
    6. Toxoplasmosis B58
    7. Varicella (Chicken Pox) B01
    8. Viral Hepatitis A B15
    9. Zika virus A928
13. Drug poisoning T360-T509

# Appendix S3 – Analysis plan for calculation of interventional disparity measures (IDMs)

We calculate the following IDMs:

- Total Adjusted Association: the total association between maternal SES and preterm birth, adjusted for confounders and standardised to the distribution of confounders.
- IDM Direct Path: the direct association between maternal SES on preterm birth that does not go through our mediators. Path 1 in DAG (figure 1). Incorporates potential conditionality between mediators.
- IDM Indirect Path through smoking: the association of maternal SES and preterm birth through smoking and through smoking and obstetric conditions. Path 2 in DAG (figure 1).
- IDM Indirect Path through Mental Ill Health: the association of maternal SES and preterm birth through mental ill health and through mental ill health and obstetric conditions. Path 3 in DAG (figure 1).
- IDM Indirect Path through Physical Health: the association of maternal SES and preterm birth through physical health and through physical health and obstetric conditions. Path 4 in DAG (figure 1).
- IDM Indirect Path through Obstetric Conditions: the association of maternal SES and preterm birth through obstetric conditions. Path 5 in DAG (figure 1).
- IDM Mediated Dependence: the path mediated through the dependence between the non-sequential mediators (the conditional dependence of physical health, mental ill health and smoking). This path is only relevant if the conditional dependence changes according to exposure status.

Using counterfactual notification, the formulae below identify how we calculate IDM Direct Paths (IDM-DE), IDM Indirect Path (IDM-IE), IDM Mediated Dependence (IDM-MD) and the adjusted total association (TAA). X is our exposure of interest (maternal SES), Y is our outcome (preterm birth, PTB), M is our mediator, and C are the baseline confounders (maternal age, marital status and parity).

The first step is to create the following models (‘richly specified’) which include confounder-SES, confounder-mediator, and confounder-confounder interaction terms.

Equation 0‑1: Logistic Model Total Adjusted Association – Outcome: Preterm Birth (PTB), exposure (X): most deprived WIMD quintile, confounders (C).

$$\log\left( \frac{p \left( \mathrm{PTB} \right)|X,C}{\left( 1-p \left( \mathrm{PTB} \right) \right)|X,C} \right)= \beta_{0}+ \beta_{1}X+\beta_{2}C+ \beta_{3}X*C+ \beta_{4}C*C$$

Equation 0‑2: Logistic Model 1 – Outcome: maternal smoking during pregnancy, exposure (X): most deprived WIMD quintile, confounders (C).

$$\log\left( \frac{p \left( \mathrm{smoking} \right)|X,C}{\left( 1-p \left( \mathrm{smoking} \right) \right)|X,C} \right)= \beta_{0}+ \beta_{1}X+\beta_{2}C+ \beta_{3}X*C+ \beta_{4}C*C$$

Equation 0‑3: Logistic Model 2* – Outcome: maternal mental ill health during pregnancy, exposure (X): most deprived WIMD quintile, confounders (C).

$$\log\left( \frac{p \left( mental health \right)|X,C}{\left( 1-p \left( mental health \right) \right)|X,C} \right)= \beta_{0}+ \beta_{1}X+\beta_{2}C+ \beta_{3}X*C+ \beta_{4}C*C$$

Equation 0‑4: Logistic Model 2’* – Outcome: maternal mental ill health during pregnancy, exposure (X): most deprived WIMD quintile, confounders (C), mediator 1 (M1): maternal smoking during pregnancy.

$$\log\left( \frac{p \left( mental health \right)|X,C,M_{1}}{\left( 1-p \left( mental health \right) \right)|X,C,M_{1}} \right)= \beta_{0}+ \beta_{1}X +\beta_{2}M_{1}+\beta_{3}C+ \beta_{4}X*M_{1}+ \beta_{5}X*C+ \beta_{6}M_{1}*C+ \beta_{7}C*C$$

*both logistic models for mental health did not include an interaction term between age and parity due to instability of the coefficients for this term.

Equation 0‑5: Logistic Model 3 – Outcome: maternal physical health issues during pregnancy, exposure (X): most deprived WIMD quintile, confounders (C).

$$\log\left( \frac{p \left( physical health \right)|X,C}{\left( 1-p \left( physical health \right) \right)|X,C} \right)= \beta_{0}+ \beta_{1}X+\beta_{2}C+ \beta_{3}X*C+ \beta_{4}C*C$$

Equation 0‑6: Logistic Model 3’ – Outcome: maternal physical health issues during pregnancy, exposure (X): most deprived WIMD quintile, confounders (C), mediator 1 (M1): maternal smoking during pregnancy, mediator 2 (M2): maternal mental ill health during pregnancy.

$\log\left( \frac{p \left( physical health \right)|X,C,M_{1},M_{2}}{\left( 1-p \left( physical health \right) \right)|X,C,M_{1},M_{2}} \right)= \beta_{0}+ \beta_{1}X +\beta_{2}M_{1}+\beta_{3}M_{2}+\beta_{4}C+ \beta_{5}X*M_{1}+\beta_{6}X*M_{2}+ \beta_{7}M_{1}*M_{2}+ \beta_{8}X*C+ \beta_{9}M_{1}*C+ \beta_{10}M_{2}*C+ \beta_{11}C*$C

Equation 0‑7: Logistic Model 4 – Outcome: maternal obstetric conditions, exposure (X): most deprived WIMD quintile, confounders (C), mediator 1 (M1): maternal smoking during pregnancy, mediator 2 (M2): maternal mental ill health during pregnancy, mediator 3 (M3): maternal physical health issues during pregnancy.

$\log\left( \frac{p \left( obstetric conditions \right)|X,C,M_{1},M_{2},M_{3}}{\left( 1-p \left( obstetric conditions \right) \right)|X,C,M_{1},M_{2},M_{3}} \right)= \beta_{0}+ \beta_{1}X +\beta_{2}M_{1}+\beta_{3}M_{2}+\beta_{4}M_{3}+\beta_{5}C+ \beta_{6}X*M_{1}+\beta_{7}X*M_{2}+\beta_{8}X*M_{3}+ \beta_{9}M_{1}*M_{2}+ \beta_{10}M_{1}*M_{3}+ \beta_{11}M_{2}*M_{3}+ \beta_{12}X*C+ \beta_{13}M_{1}*C+ \beta_{14}M_{2}*C+ \beta_{15}M_{3}*C+ \beta_{16}C*$C

Equation 0‑8: Logistic Model 5 – Outcome: Preterm Birth (PTB), exposure (X): most deprived WIMD quintile, confounders (C), mediator 1 (M1): maternal smoking during pregnancy, mediator 2 (M2): maternal mental ill health during pregnancy, mediator 3 (M3): maternal physical health issues during pregnancy, mediator 4 (M4): maternal obstetric conditions.

$\log\left( \frac{p \left( \mathrm{PTB} \right)|X,C,M_{1},M_{2},M_{3},M_{4}}{\left( 1-p \left( \mathrm{PTB} \right) \right)|X,C,M_{1},M_{2},M_{3},M_{4}} \right)= \beta_{0}+ \beta_{1}X +\beta_{2}M_{1}+\beta_{3}M_{2}+\beta_{4}M_{3}+\beta_{5}C+ \beta_{6}X*M_{1}+\beta_{7}X*M_{2}+\beta_{8}X*M_{3}+ \beta_{9}M_{1}*M_{2}+ \beta_{10}M_{1}*M_{3}+ \beta_{11}M_{2}*M_{3}+ \beta_{12}X*C+ \beta_{13}M_{1}*C+ \beta_{14}M_{2}*C+ \beta_{15}M_{3}*C+\beta_{16}M_{4}+{\beta_{17}X*M_{4}+\beta_{18}M_{1}*M_{4}+ \beta_{19}M_{2}*M_{4}+ \beta_{20}M_{3}*M_{4}+\beta_{21}M_{4}*C+\beta}_{22}C*$C

These models are used to estimate the following terms:

- Equation 0‑2, Equation 0‑3 and Equation 0‑5 are used to take a random draw from the distribution for each mediator when WIMD quintile is set to least deprived (M1^0^_C_, M2^0^_C_, M3^0^_C_) and most deprived (M1^1^_C_, M2^1^_C_, M3^1^_C_), marginal to other mediators.
- Equation 0‑4 and Equation 0‑6 are used to take a random draw from the distribution for M2’ ­_­_ (M2 conditional on M1), and M3’ (M3 conditional on M1 and M2’). Combined with M1^0^ these give me a joint distribution for mediators, so M^0^_c_ = (M1^0^_C_, M2’^0^_C_, M3’^0^_C_, M4^0^_C_(M1^0^_C_, M2’^0^_C_, M3’^0^_C_)).
- Equation 0‑7 is used to take a random draw from the distribution for mediator 4 - M4^0^_C_(M1^0^_C_, M2^0^_C_, M3^0^_C_), M4^0^_C_(M1^1^_C_, M2^0^_C_, M3^0^_C_), M4^0^_C_(M1^1^_C_, M2^1^_C_, M3^0^_C_), M4^0^_C_(M1^1^_C_, M2^1^_C_, M3^1^_C_) and M4^1^_C_(M1^1^_C_, M2^1^_C_, M3^1^_C_). I would first estimate the distribution of M1, M2 and M3 as detailed above, then use these draws with the WIMD exposure setting as the input for Model 4.

Due to the complexity of the pathway (4 mediators, both ordered and not) I have decided to calculate mediator specific effects.^4^ I have defined the mediated paths as the effect through smoking including the effect of smoking on obstetric conditions (path 2), through maternal mental health including the effect of maternal mental health on obstetric conditions (path 3), through maternal physical health including the effect of maternal physical health on obstetric conditions (path 4), and through obstetric conditions alone (path 5).

Monte Carlo Simulations (at 200 expansion for each imputation in the imputed analysis and 300 for complete case to remove Monte Carlo error) are conducted to create the following estimates from the simulated datasets:

1. P[Y(X^1^_c_)|C=c] - use Equation 0‑1 to estimate proportion with preterm birth when SES is set to most deprived quintile.
2. P[Y(X^0^_c_)|C=c] - use Equation 0‑1 to estimate proportion with preterm birth when SES is set to least deprived quintile.
3. P[Y(X^1^_c_M^0^_c_)|C=c] - use Equation 0‑2, Equation 0‑4, Equation 0‑6 and Equation 0‑7 to estimate mediator distribution when SES set to least deprived quintile conditional on other mediators as described, then set SES to most deprived quintile and use Equation 0‑8 to estimate proportion with preterm birth.
4. P[Y(X^0^_c_M^0^_c_)|C=c] - use Equation 0‑2, Equation 0‑4, Equation 0‑6 and Equation 0‑7 to estimate mediator distribution when SES set to least deprived quintile conditional on other mediators as described, then set SES to least deprived quintile and use Equation 0‑8 to estimate proportion with preterm birth.
5. P[Y(X^1^_c_M1^1^_c_M2^0^_c_M3^0^_c_M4^0^_C_(M1^1^_C_, M2^0^_C_, M3^0^_C_) )|C=c] - use Equation 0‑2 to estimate smoking distribution when SES set to most deprived quintile, use Equation 0‑3 to estimate mental ill health distribution when SES set to least deprived quintile, use Equation 0‑5 to estimate physical health distribution when SES set to least deprived quintile, use Equation 0‑7 to estimate obstetric condition distribution when SES set to least deprived quintile conditional on other mediators. Set SES to most deprived quintile then use Equation 0‑8 to estimate proportion with preterm birth.
6. P[Y(X^1^_c_M1^0^_c_M2^0^_c_M3^0^_c_M4^0^_C_(M1^0^_C_, M2^0^_C_, M3^0^_C_) )|C=c] - use Equation 0‑2 to estimate smoking distribution when SES set to least deprived quintile, use Equation 0‑3 to estimate mental ill health distribution when SES set to least deprived quintile, use Equation 0‑5 to estimate physical health distribution when SES set to least deprived quintile, use Equation 0‑7 to estimate obstetric condition distribution when SES set to least deprived quintile conditional on other mediators. Set SES to most deprived quintile then use Equation 0‑8 to estimate proportion with preterm birth.
7. P[Y(X^1^_c_M1^1^_c_M2^1^_c_M3^0^_c_M4^0^_C_(M1^1^_C_, M2^1^_C_, M3^0^_C_) )|C=c] - use Equation 0‑2 to estimate smoking distribution when SES set to most deprived quintile, use Equation 0‑3 to estimate mental ill health distribution when SES set to most deprived quintile, use Equation 0‑5 to estimate physical health distribution when SES set to least deprived quintile, use Equation 0‑7 to estimate obstetric condition distribution when SES set to least deprived quintile conditional on other mediators. Set SES to most deprived quintile then use Equation 0‑8 to estimate proportion with preterm birth.
8. P[Y(X^1^_c_M1^1^_c_M2^1^_c_M3^1^_c_M4^0^_C_(M1^1^_C_, M2^1^_C_, M3^1^_C_) )|C=c] - use Equation 0‑2 to estimate smoking distribution when SES set to most deprived quintile, use Equation 0‑3 to estimate mental ill health distribution when SES set to most deprived quintile, use Equation 0‑5 to estimate physical health distribution when SES set to most deprived quintile, use Equation 0‑7 to estimate obstetric condition distribution when SES set to least deprived quintile conditional on other mediators. Set SES to most deprived quintile then use Equation 0‑8 to estimate proportion with preterm birth.
9. P[Y(X^1^_c_M1^1^_c_M2^1^_c_M3^1^_c_M4^1^_C_(M1^1^_C_, M2^1^_C_, M3^1^_C_) )|C=c] - use Equation 0‑2 to estimate smoking distribution when SES set to most deprived quintile, use Equation 0‑3 to estimate mental ill health distribution when SES set to most deprived quintile, use Equation 0‑5 to estimate physical health distribution when SES set to most deprived quintile, use Equation 0‑7 to estimate obstetric condition distribution when SES set to most deprived quintile conditional on other mediators. Set SES to most deprived quintile then use Equation 0‑8 to estimate proportion with preterm birth.

Using the outputs for PTB prevalence from my simulated datasets, IDMs are:

TAA = P[Y(X^1^_c_)|C=c] - P[Y(X^0^_c_)|C=c]

IDM-DE = P[Y(X^1^_c_M^0^_c_)|C=c]- P[Y(X^0^_c_M^0^_c_)|C=c]

IDM-IE1 = P[Y(X^1^_c_M1^1^_c_M2^0^_c_M3^0^_c_M4^0^_C_(M1^1^_C_, M2^0^_C_, M3^0^_C_) )|C=c] – P[Y(X^1^_c_M1^0^_c_M2^0^_c_M3^0^_c_M4^0^_C_(M1^0^_C_, M2^0^_C_, M3^0^_C_) )|C=c]

IDM-IE2 = P[Y(X^1^_c_M1^1^_c_M2^1^_c_M3^0^_c_M4^0^_C_(M1^1^_C_, M2^1^_C_, M3^0^_C_) )|C=c] – P[Y(X^1^_c_M1^1^_c_M2^0^_c_M3^0^_c_M4^0^_C_(M1^1^_C_, M2^0^_C_, M3^0^_C_) )|C=c]

IDM-IE3 = P[Y(X^1^_c_M1^1^_c_M2^1^_c_M3^1^_c_M4^0^_C_(M1^1^_C_, M2^1^_C_, M3^1^_C_) )|C=c] – P[Y(X^1^_c_M1^1^_c_M2^1^_c_M3^0^_c_M4^0^_C_(M1^1^_C_, M2^1^_C_, M3^0^_C_) )|C=c]

IDM-IE4 = P[Y(X^1^_c_M1^1^_c_M2^1^_c_M3^1^_c_M4^1^_C_(M1^1^_C_, M2^1^_C_, M3^1^_C_) )|C=c] – P[Y(X^1^_c_M1^1^_c_M2^1^_c_M3^1^_c_M4^0^_C_(M1^1^_C_, M2^1^_C_, M3^1^_C_) )|C=c]

IDM-MD = TAA - IDM-DE - IDM-IE1 - IDM-IE2 - IDM-IE3 - IDM-IE4

# Appendix S4 – Pattern of missing data

Pattern of missing data in dataset, with blue cells representing variables where data are not missing, and red cells representing variables where data are missing. Top row – variable name, Left axis – number of cases which have this pattern of missing data, Right axis – number of variables missing, Bottom row – total number of cases with specific variables missing.

# Appendix S5 – Inequality in preterm birth and mediators

Figure A: Distribution of preterm births in the cohort across quintiles of Welsh Index of Multiple Deprivation (IMD). Y axis is percentage of total births which are preterm.

Figure B: Distribution of smoking during pregnancy in the cohort across quintiles of Welsh Index of Multiple Deprivation (IMD). Y axis is percentage of total births.

Figure C: Distribution of maternal mental ill health in the cohort across quintiles of Welsh Index of Multiple Deprivation (IMD). Y axis is percentage of total births.

Figure D: Distribution of maternal physical health conditions in the cohort across quintiles of Welsh Index of Multiple Deprivation (IMD). Y axis is percentage of total births.

Figure E: Distribution of maternal obstetric conditions in the cohort across quintiles of Welsh Index of Multiple Deprivation (IMD). Y axis is percentage of total births.

# Appendix S6 – Distribution of excluded data

|  | | N | % |
| --- | --- | --- | --- |
| WIMD | 1 | 9096 | 5.0 |
|  | 2 | 8044 | 4.5 |
|  | 3 | 7517 | 4.2 |
|  | 4 | 7873 | 4.4 |
|  | 5 | 7592 | 4.2 |
|  | Missing | 140256 | 77.8 |
| Preterm Birth | No | 56785 | 31.5 |
|  | Yes | 16805 | 9.3 |
|  | Missing or Out of range | 106788 | 59.2 |
| Smoking During Pregnancy | No | 25365 | 14.1 |
|  | Yes | 6408 | 3.6 |
|  | Missing | 148605 | 82.4 |
| Maternal Mental Health | No | 178233 | 98.8 |
|  | Yes | 2145 | 1.2 |
| Maternal Physical Health | No | 174235 | 96.6 |
|  | Yes | 6143 | 3.4 |
| Obstetric Conditions | No | 167840 | 93.0 |
|  | Yes | 12538 | 7.0 |
| Maternal Age | <18 | 2922 | 1.6 |
|  | 18-24 | 30906 | 17.1 |
|  | 25-34 | 68789 | 38.1 |
|  | 35+ | 23963 | 13.3 |
|  | Missing or Out of range | 53798 | 29.8 |
| Parity | 0 | 27805 | 15.4 |
|  | 1 | 16458 | 9.1 |
|  | 2+ | 11590 | 6.4 |
|  | Missing or Out of range | 124525 | 69.0 |
| Marital Status | Married/civil partnership | 25949 | 14.4 |
|  | Not married, sole registration | 4498 | 2.5 |
|  | Not married, joint registration, parents same address | 16670 | 9.2 |
|  | Not married, joint registration, parents different address | 5132 | 2.8 |
|  | Missing | 128129 | 71.0 |

# Appendix S7 – Distribution of variables by year (preterm birth (PTB) for full cohort, mediators for complete case)

|  | PTB | Smoker | Mental Health | Physical Health | Obstetric Conditions |
| --- | --- | --- | --- | --- | --- |
| 2000 | 6.07 | 22.48 | 1.26 | 7.44 | 24.29 |
| 2001 | 5.98 | 23.51 | 2.10 | 8.96 | 25.66 |
| 2002 | 6.48 | 24.07 | 2.71 | 9.34 | 24.68 |
| 2003 | 6.68 | 22.46 | 2.99 | 8.15 | 24.12 |
| 2004 | 6.52 | 25.13 | 3.54 | 8.99 | 23.06 |
| 2005 | 5.98 | 24.26 | 3.76 | 9.91 | 21.70 |
| 2006 | 6.08 | 23.81 | 3.63 | 8.55 | 20.85 |
| 2007 | 6.21 | 23.94 | 3.56 | 8.25 | 20.85 |
| 2008 | 5.84 | 22.82 | 3.36 | 9.25 | 21.05 |
| 2009 | 5.98 | 23.29 | 3.63 | 10.19 | 22.35 |
| 2010 | 5.79 | 23.20 | 3.50 | 10.74 | 23.18 |
| 2011 | 5.87 | 23.95 | 3.72 | 11.55 | 23.01 |
| 2012 | 5.62 | 24.50 | 4.20 | 11.38 | 23.56 |
| 2013 | 5.66 | 24.85 | 4.13 | 11.65 | 23.94 |
| 2014 | 5.73 | 21.86 | 4.60 | 12.84 | 23.71 |
| 2015 | 5.74 | 20.68 | 4.95 | 12.55 | 23.44 |
| 2016 | 6.16 | 21.10 | 5.46 | 12.51 | 22.73 |
| 2017 | 6.43 | 19.57 | 5.97 | 13.29 | 22.78 |
| 2018 | 6.49 | 19.12 | 6.09 | 14.64 | 22.88 |
| 2019 | 6.65 | 18.86 | 6.72 | 17.36 | 24.84 |

# Appendix S8 – Full regression outputs for CDE estimates

Table A: Model results and 95% confidence intervals for total effects logistic non-mediator adjusted regressions (exposure and confounders), for preterm birth (<37 weeks)

|  |  | AOR | LCL | UCL | p-value |
| --- | --- | --- | --- | --- | --- |
| Socioeconomic Status | 1 | 1.26 | 1.22 | 1.31 | <0.0005 |
|  | 2 | 1.18 | 1.13 | 1.22 | <0.0005 |
|  | 3 | 1.10 | 1.06 | 1.15 | <0.0005 |
|  | 4 | 1.06 | 1.02 | 1.10 | 0.006 |
|  | 5 | REF | . | . | . |
| Maternal Age | <18 | 1.13 | 1.05 | 1.21 | 0.001 |
|  | 18-24 | REF | . | . | . |
|  | 25-34 | 1.04 | 1.01 | 1.07 | 0.004 |
|  | 35+ | 1.21 | 1.17 | 1.26 | <0.0005 |
| Marital Status | married/civil partnership | REF | . | . | . |
|  | Not married, sole registration | 1.42 | 1.36 | 1.48 | <0.0005 |
|  | Not married, joint registration, parents same address | 1.12 | 1.09 | 1.14 | <0.0005 |
|  | Not married, joint registration, parents different address | 1.31 | 1.26 | 1.36 | <0.0005 |
| Parity | 0 | REF | . | . | . |
|  | 1 | 0.75 | 0.73 | 0.77 | <0.0005 |
|  | 2+ | 1.04 | 1.01 | 1.07 | 0.019 |

Table B: Model results and 95% confidence intervals for total effects logistic mediator adjusted regressions (exposure, mediators and confounders), for preterm birth (<37 weeks).

|  |  | AOR | LCL | UCL | p-value |
| --- | --- | --- | --- | --- | --- |
| Socioeconomic Status | 1 | 1.21 | 1.14 | 1.27 | <0.0005 |
|  | 2 | 1.14 | 1.08 | 1.20 | <0.0005 |
|  | 3 | 1.08 | 1.02 | 1.14 | 0.006 |
|  | 4 | 1.05 | 0.99 | 1.11 | 0.085 |
|  | 5 | REF | . | . | . |
| Smoking During Pregnancy | No | REF | . | . | . |
|  | Yes | 1.45 | 1.30 | 1.61 | <0.0005 |
| Maternal Mental Health | No | REF | . | . | . |
|  | Yes | 1.54 | 1.31 | 1.80 | <0.0005 |
| Maternal Physical Health | No | REF | . | . | . |
|  | Yes | 1.76 | 1.62 | 1.92 | <0.0005 |
| Obstetric Conditions | No | REF | . | . | . |
|  | Yes | 2.14 | 2.01 | 2.28 | <0.0005 |
| Maternal Age | <18 | 1.13 | 1.06 | 1.21 | <0.0005 |
|  | 18-24 | REF | . | . | . |
|  | 25-34 | 1.11 | 1.08 | 1.14 | <0.0005 |
|  | 35+ | 1.30 | 1.25 | 1.35 | <0.0005 |
| Marital Status | married/civil partnership | REF | . | . | . |
|  | Not married, sole registration | 1.19 | 1.14 | 1.24 | <0.0005 |
|  | Not married, joint registration, parents same address | 1.04 | 1.01 | 1.07 | 0.002 |
|  | Not married, joint registration, parents different address | 1.14 | 1.10 | 1.19 | <0.0005 |
| Parity | 0 | REF | . | . | . |
|  | 1 | 0.74 | 0.72 | 0.76 | <0.0005 |
|  | 2+ | 0.97 | 0.94 | 1.00 | 0.070 |
| Interaction terms | WIMD 1:Smoker | 1.06 | 0.94 | 1.20 | 0.334 |
|  | WIMD 2:Smoker | 1.06 | 0.95 | 1.19 | 0.272 |
|  | WIMD 3:Smoker | 1.08 | 0.95 | 1.23 | 0.212 |
|  | WIMD 4:Smoker | 1.03 | 0.91 | 1.18 | 0.618 |
|  | WIMD 1:MH | 0.95 | 0.80 | 1.13 | 0.567 |
|  | WIMD 2:MH | 0.89 | 0.75 | 1.06 | 0.204 |
|  | WIMD 3:MH | 0.85 | 0.70 | 1.02 | 0.081 |
|  | WIMD 4:MH | 1.04 | 0.85 | 1.26 | 0.696 |
|  | WIMD 1:PH | 0.89 | 0.81 | 0.98 | 0.021 |
|  | WIMD 2:PH | 0.98 | 0.89 | 1.08 | 0.687 |
|  | WIMD 3:PH | 1.01 | 0.91 | 1.11 | 0.922 |
|  | WIMD 4:PH | 1.04 | 0.94 | 1.16 | 0.421 |
|  | WIMD 1:OC | 0.93 | 0.86 | 1.00 | 0.062 |
|  | WIMD 2:OC | 0.94 | 0.87 | 1.02 | 0.121 |
|  | WIMD 3:OC | 0.97 | 0.90 | 1.05 | 0.500 |
|  | WIMD 4:OC | 0.97 | 0.89 | 1.06 | 0.520 |
|  | Smoker:MH | 0.97 | 0.87 | 1.08 | 0.570 |
|  | PH:MH | 0.98 | 0.87 | 1.09 | 0.676 |
|  | Smoker:PH | 0.92 | 0.85 | 0.98 | 0.017 |
|  | PH:OC | 0.86 | 0.81 | 0.91 | <0.0005 |
|  | MH:OC | 0.88 | 0.80 | 0.97 | 0.013 |
|  | Smoker:OC | 0.91 | 0.85 | 0.97 | 0.002 |

MH – Maternal Mental Health, PH – Maternal Physical Health, OC – Obstetric Conditions

Table C: Model results and 95% confidence intervals for total effects logistic non-mediator adjusted regressions (exposure and confounders), with each gestational age category compared to term (between 39 weeks and 41^+6^ weeks).

|  |  | Extremely PTB | | | | Very PTB | | | | Moderate PTB | | | | Early Term | | | | Post term | | | |
| --- | --- | --- | --- | --- | --- | --- | --- | --- | --- | --- | --- | --- | --- | --- | --- | --- | --- | --- | --- | --- | --- |
|  |  | AOR | UCL | LCL | p value | AOR | UCL | LCL | p value | AOR | UCL | LCL | p value | AOR | UCL | LCL | p value | AOR | UCL | LCL | p value |
| Socioeconomic Status | 1 | 1.44 | 1.23 | 1.69 | <0.0005 | 1.30 | 1.17 | 1.45 | <0.0005 | 1.29 | 1.24 | 1.34 | <0.0005 | 1.17 | 1.14 | 1.19 | <0.0005 | 0.99 | 0.95 | 1.03 | 0.571 |
|  | 2 | 1.18 | 1.00 | 1.40 | 0.053 | 1.24 | 1.11 | 1.38 | <0.0005 | 1.20 | 1.15 | 1.25 | <0.0005 | 1.13 | 1.11 | 1.16 | <0.0005 | 0.96 | 0.92 | 1.00 | 0.036 |
|  | 3 | 1.12 | 0.94 | 1.32 | 0.214 | 1.12 | 1.00 | 1.25 | 0.053 | 1.12 | 1.08 | 1.17 | <0.0005 | 1.09 | 1.07 | 1.12 | <0.0005 | 0.99 | 0.95 | 1.03 | 0.512 |
|  | 4 | 1.23 | 1.03 | 1.46 | 0.019 | 1.05 | 0.94 | 1.18 | 0.373 | 1.05 | 1.01 | 1.10 | 0.020 | 1.03 | 1.01 | 1.06 | 0.006 | 0.97 | 0.93 | 1.01 | 0.112 |
|  | 5 | REF | . | . | . | REF | . | . | . | REF | . | . | . | REF | . | . | . | REF | . | . | . |
| Maternal Age | <18 | 1.44 | 1.13 | 1.84 | 0.003 | 1.17 | 0.97 | 1.41 | 0.100 | 1.09 | 1.01 | 1.18 | 0.025 | 1.02 | 0.97 | 1.07 | 0.504 | 0.89 | 0.81 | 0.97 | 0.005 |
|  | 18-24 | REF | . | . | . | REF | . | . | . | REF | . | . | . | REF | . | . | . | REF | . | . | . |
|  | 25-34 | 1.01 | 0.90 | 1.14 | 0.844 | 1.07 | 0.99 | 1.16 | 0.090 | 1.06 | 1.02 | 1.09 | <0.0005 | 1.04 | 1.02 | 1.06 | <0.0005 | 1.05 | 1.01 | 1.08 | 0.006 |
|  | 35+ | 1.15 | 0.98 | 1.36 | 0.087 | 1.36 | 1.22 | 1.51 | <0.0005 | 1.27 | 1.22 | 1.32 | <0.0005 | 1.23 | 1.20 | 1.25 | <0.0005 | 1.04 | 1.00 | 1.09 | 0.057 |
| Marital Status | married/civil partnership | REF | . | . | . | REF | . | . | . | REF | . | . | . | REF | . | . | . | REF | . | . | . |
|  | Not married, sole registration | 1.30 | 1.08 | 1.58 | 0.007 | 1.65 | 1.47 | 1.86 | <0.0005 | 1.44 | 1.37 | 1.50 | <0.0005 | 1.11 | 1.08 | 1.14 | <0.0005 | 1.05 | 1.00 | 1.11 | 0.064 |
|  | Not married, joint registration, parents same address | 1.19 | 1.06 | 1.35 | 0.004 | 1.18 | 1.10 | 1.28 | <0.0005 | 1.10 | 1.07 | 1.13 | <0.0005 | 0.98 | 0.96 | 0.99 | 0.006 | 1.05 | 1.02 | 1.08 | 0.003 |
|  | Not married, joint registration, parents different address | 1.75 | 1.51 | 2.03 | <0.0005 | 1.51 | 1.36 | 1.67 | <0.0005 | 1.28 | 1.24 | 1.34 | <0.0005 | 1.07 | 1.05 | 1.10 | <0.0005 | 1.01 | 0.96 | 1.05 | 0.730 |
| Parity | 0 | REF | . | . | . | REF | . | . | . | REF | . | . | . | REF | . | . | . | REF | . | . | . |
|  | 1 | 0.53 | 0.47 | 0.61 | <0.0005 | 0.62 | 0.57 | 0.68 | <0.0005 | 0.77 | 0.74 | 0.79 | <0.0005 | 1.09 | 1.07 | 1.11 | <0.0005 | 0.53 | 0.51 | 0.55 | <0.0005 |
|  | 2+ | 0.94 | 0.81 | 1.09 | 0.401 | 0.95 | 0.86 | 1.04 | 0.245 | 1.11 | 1.08 | 1.15 | <0.0005 | 1.41 | 1.38 | 1.43 | <0.0005 | 0.55 | 0.53 | 0.57 | <0.0005 |

Table D: Model results and 95% confidence intervals for total effects logistic mediator adjusted regressions (exposure, mediators and confounders), with each gestational age category compared to term (between 39 weeks and 41^+6^ weeks).

|  |  | Extremely PTB | | | | Very PTB | | | | Moderate PTB | | | | Early Term | | | | Post term | | | |
| --- | --- | --- | --- | --- | --- | --- | --- | --- | --- | --- | --- | --- | --- | --- | --- | --- | --- | --- | --- | --- | --- |
|  |  | AOR | UCL | LCL | p value | AOR | UCL | LCL | p value | AOR | UCL | LCL | p value | AOR | UCL | LCL | p value | AOR | UCL | LCL | p value |
| Socioeconomic Status | 1 | 1.16 | 0.90 | 1.49 | 0.240 | 1.34 | 1.14 | 1.58 | <0.0005 | 1.22 | 1.15 | 1.30 | <0.0005 | 1.13 | 1.10 | 1.16 | <0.0005 | 1.03 | 0.98 | 1.08 | 0.216 |
|  | 2 | 1.03 | 0.78 | 1.34 | 0.853 | 1.26 | 1.06 | 1.48 | 0.007 | 1.16 | 1.09 | 1.22 | <0.0005 | 1.11 | 1.07 | 1.14 | <0.0005 | 0.98 | 0.93 | 1.04 | 0.546 |
|  | 3 | 1.01 | 0.78 | 1.31 | 0.940 | 1.14 | 0.97 | 1.35 | 0.114 | 1.10 | 1.03 | 1.16 | 0.002 | 1.07 | 1.03 | 1.10 | <0.0005 | 1.01 | 0.96 | 1.06 | 0.670 |
|  | 4 | 1.21 | 0.93 | 1.57 | 0.153 | 1.04 | 0.87 | 1.24 | 0.654 | 1.05 | 0.99 | 1.11 | 0.131 | 1.02 | 0.99 | 1.05 | 0.175 | 0.97 | 0.92 | 1.02 | 0.279 |
|  | 5 | REF | . | . | . | REF | . | . | . | REF | . | . | . | REF | . | . | . | REF | . | . | . |
| Smoking During Pregnancy | No | REF | . | . | . | REF | . | . | . | REF | . | . | . | REF | . | . | . | REF | . | . | . |
|  | Yes | 1.76 | 1.18 | 2.63 | 0.006 | 1.64 | 1.24 | 2.18 | 0.001 | 1.48 | 1.33 | 1.64 | <0.0005 | 1.21 | 1.13 | 1.29 | <0.0005 | 1.04 | 0.92 | 1.16 | 0.550 |
| Maternal Mental Health | No | REF | . | . | . | REF | . | . | . | REF | . | . | . | REF | . | . | . | REF | . | . | . |
|  | Yes | 1.91 | 0.98 | 3.70 | 0.056 | 1.68 | 1.07 | 2.63 | 0.023 | 1.55 | 1.30 | 1.84 | <0.0005 | 1.20 | 1.08 | 1.34 | 0.001 | 0.94 | 0.76 | 1.16 | 0.562 |
| Maternal Physical Health | No | REF | . | . | . | REF | . | . | . | REF | . | . | . | REF | . | . | . | REF | . | . | . |
|  | Yes | 1.55 | 1.03 | 2.34 | 0.036 | 1.75 | 1.35 | 2.27 | <0.0005 | 1.95 | 1.78 | 2.14 | <0.0005 | 1.49 | 1.41 | 1.58 | <0.0005 | 1.22 | 1.09 | 1.37 | <0.0005 |
| Obstetric Conditions | No | REF | . | . | . | REF | . | . | . | REF | . | . | . | REF | . | . | . | REF | . | . | . |
|  | Yes | 3.39 | 2.58 | 4.47 | <0.0005 | 3.50 | 2.93 | 4.19 | <0.0005 | 2.21 | 2.06 | 2.37 | <0.0005 | 1.80 | 1.73 | 1.87 | <0.0005 | 0.81 | 0.75 | 0.89 | <0.0005 |
| Maternal Age | <18 | 1.42 | 1.11 | 1.81 | 0.005 | 1.17 | 0.97 | 1.41 | 0.101 | 1.10 | 1.02 | 1.18 | 0.018 | 1.02 | 0.97 | 1.07 | 0.437 | 0.89 | 0.81 | 0.97 | 0.005 |
|  | 18-24 | REF | . | . | . | REF | . | . | . | REF | . | . | . | REF | . | . | . | REF | . | . | . |
|  | 25-34 | 1.14 | 1.01 | 1.28 | 0.035 | 1.18 | 1.08 | 1.28 | <0.0005 | 1.13 | 1.09 | 1.16 | <0.0005 | 1.08 | 1.06 | 1.10 | <0.0005 | 1.04 | 1.01 | 1.07 | 0.023 |
|  | 35+ | 1.32 | 1.12 | 1.56 | 0.001 | 1.51 | 1.36 | 1.68 | <0.0005 | 1.37 | 1.31 | 1.42 | <0.0005 | 1.28 | 1.25 | 1.31 | <0.0005 | 1.04 | 0.99 | 1.08 | 0.113 |
| Marital Status | married/civil partnership | REF | . | . | . | REF | . | . | . | REF | . | . | . | REF | . | . | . | REF | . | . | . |
|  | Not married, sole registration | 0.95 | 0.78 | 1.15 | 0.602 | 1.29 | 1.14 | 1.46 | <0.0005 | 1.20 | 1.14 | 1.25 | <0.0005 | 1.00 | 0.97 | 1.03 | 0.920 | 1.07 | 1.01 | 1.13 | 0.015 |
|  | Not married, joint registration, parents same address | 1.05 | 0.93 | 1.19 | 0.449 | 1.08 | 1.00 | 1.17 | 0.062 | 1.03 | 1.00 | 1.06 | 0.076 | 0.94 | 0.93 | 0.96 | <0.0005 | 1.05 | 1.02 | 1.08 | 0.002 |
|  | Not married, joint registration, parents different address | 1.37 | 1.18 | 1.59 | <0.0005 | 1.24 | 1.12 | 1.38 | <0.0005 | 1.11 | 1.07 | 1.16 | <0.0005 | 0.99 | 0.97 | 1.01 | 0.405 | 1.02 | 0.98 | 1.07 | 0.370 |
| Parity | 0 | REF | . | . | . | REF | . | . | . | REF | . | . | . | REF | . | . | . | REF | . | . | . |
|  | 1 | 0.53 | 0.46 | 0.60 | <0.0005 | 0.62 | 0.57 | 0.68 | <0.0005 | 0.77 | 0.74 | 0.79 | <0.0005 | 1.09 | 1.07 | 1.11 | <0.0005 | 0.53 | 0.51 | 0.54 | <0.0005 |
|  | 2+ | 0.86 | 0.74 | 0.99 | 0.038 | 0.89 | 0.81 | 0.98 | 0.016 | 1.05 | 1.02 | 1.09 | 0.002 | 1.37 | 1.34 | 1.40 | <0.0005 | 0.55 | 0.53 | 0.58 | <0.0005 |
| Interaction terms | WIMD 1:Smoker | 1.27 | 0.83 | 1.94 | 0.269 | 1.04 | 0.77 | 1.39 | 0.809 | 1.06 | 0.94 | 1.21 | 0.322 | 1.06 | 0.98 | 1.14 | 0.145 | 0.88 | 0.79 | 0.99 | 0.033 |
|  | WIMD 2:Smoker | 1.33 | 0.83 | 2.13 | 0.233 | 1.06 | 0.78 | 1.43 | 0.717 | 1.04 | 0.93 | 1.17 | 0.464 | 1.01 | 0.94 | 1.09 | 0.712 | 0.90 | 0.78 | 1.04 | 0.167 |
|  | WIMD 3:Smoker | 1.26 | 0.78 | 2.05 | 0.344 | 0.98 | 0.70 | 1.36 | 0.881 | 1.08 | 0.95 | 1.22 | 0.239 | 1.06 | 0.98 | 1.15 | 0.129 | 0.97 | 0.85 | 1.10 | 0.645 |
|  | WIMD 4:Smoker | 1.06 | 0.63 | 1.80 | 0.815 | 0.97 | 0.69 | 1.37 | 0.861 | 1.03 | 0.91 | 1.18 | 0.630 | 1.03 | 0.95 | 1.11 | 0.494 | 0.98 | 0.85 | 1.13 | 0.767 |
|  | WIMD 1:MH | 0.67 | 0.33 | 1.39 | 0.281 | 1.01 | 0.63 | 1.62 | 0.970 | 0.99 | 0.82 | 1.19 | 0.923 | 1.08 | 0.96 | 1.22 | 0.205 | 0.90 | 0.71 | 1.15 | 0.413 |
|  | WIMD 2:MH | 1.02 | 0.49 | 2.12 | 0.961 | 0.77 | 0.46 | 1.28 | 0.311 | 0.93 | 0.76 | 1.13 | 0.475 | 1.13 | 0.99 | 1.27 | 0.062 | 0.99 | 0.77 | 1.27 | 0.919 |
|  | WIMD 3:MH | 0.83 | 0.38 | 1.84 | 0.649 | 1.05 | 0.63 | 1.75 | 0.863 | 0.84 | 0.68 | 1.04 | 0.113 | 1.09 | 0.96 | 1.24 | 0.193 | 0.89 | 0.68 | 1.16 | 0.380 |
|  | WIMD 4:MH | 1.14 | 0.52 | 2.49 | 0.748 | 1.04 | 0.60 | 1.80 | 0.899 | 1.09 | 0.88 | 1.36 | 0.423 | 1.15 | 1.00 | 1.32 | 0.050 | 1.03 | 0.78 | 1.37 | 0.821 |
|  | WIMD 1:PH | 1.01 | 0.66 | 1.57 | 0.949 | 0.94 | 0.71 | 1.25 | 0.682 | 0.88 | 0.79 | 0.98 | 0.016 | 0.98 | 0.92 | 1.05 | 0.567 | 0.93 | 0.81 | 1.07 | 0.309 |
|  | WIMD 2:PH | 1.17 | 0.74 | 1.85 | 0.498 | 1.16 | 0.87 | 1.54 | 0.320 | 0.95 | 0.85 | 1.06 | 0.330 | 0.98 | 0.91 | 1.05 | 0.508 | 0.97 | 0.84 | 1.11 | 0.647 |
|  | WIMD 3:PH | 1.14 | 0.71 | 1.83 | 0.589 | 1.25 | 0.93 | 1.68 | 0.145 | 0.97 | 0.86 | 1.08 | 0.556 | 1.00 | 0.93 | 1.07 | 0.900 | 0.83 | 0.72 | 0.96 | 0.014 |
|  | WIMD 4:PH | 1.23 | 0.76 | 1.97 | 0.397 | 1.14 | 0.84 | 1.56 | 0.405 | 1.02 | 0.91 | 1.15 | 0.701 | 0.99 | 0.92 | 1.07 | 0.810 | 0.90 | 0.77 | 1.05 | 0.177 |
|  | WIMD 1:OC | 1.06 | 0.76 | 1.47 | 0.724 | 0.72 | 0.58 | 0.89 | 0.003 | 0.94 | 0.86 | 1.02 | 0.130 | 0.91 | 0.86 | 0.95 | <0.0005 | 0.99 | 0.89 | 1.10 | 0.865 |
|  | WIMD 2:OC | 0.88 | 0.62 | 1.25 | 0.480 | 0.77 | 0.62 | 0.96 | 0.023 | 0.97 | 0.89 | 1.06 | 0.527 | 0.98 | 0.93 | 1.04 | 0.530 | 0.96 | 0.86 | 1.07 | 0.485 |
|  | WIMD 3:OC | 0.99 | 0.69 | 1.40 | 0.935 | 0.81 | 0.65 | 1.02 | 0.080 | 1.00 | 0.92 | 1.09 | 0.978 | 1.00 | 0.95 | 1.05 | 0.999 | 0.98 | 0.87 | 1.09 | 0.667 |
|  | WIMD 4:OC | 0.93 | 0.65 | 1.33 | 0.678 | 0.99 | 0.78 | 1.26 | 0.960 | 0.98 | 0.89 | 1.08 | 0.695 | 1.02 | 0.97 | 1.08 | 0.385 | 1.01 | 0.90 | 1.13 | 0.908 |
|  | Smoker:MH | 0.97 | 0.60 | 1.55 | 0.882 | 0.93 | 0.68 | 1.28 | 0.666 | 0.96 | 0.85 | 1.08 | 0.455 | 0.94 | 0.87 | 1.02 | 0.138 | 1.03 | 0.87 | 1.23 | 0.700 |
|  | PH:MH | 1.16 | 0.73 | 1.84 | 0.529 | 1.07 | 0.78 | 1.45 | 0.678 | 0.98 | 0.86 | 1.11 | 0.714 | 1.03 | 0.95 | 1.13 | 0.466 | 0.90 | 0.73 | 1.13 | 0.366 |
|  | Smoker:PH | 0.87 | 0.64 | 1.17 | 0.342 | 0.92 | 0.76 | 1.11 | 0.382 | 0.92 | 0.84 | 0.99 | 0.036 | 0.95 | 0.90 | 1.01 | 0.095 | 0.96 | 0.86 | 1.07 | 0.451 |
|  | PH:OC | 0.78 | 0.60 | 1.01 | 0.059 | 0.75 | 0.64 | 0.89 | 0.001 | 0.89 | 0.83 | 0.94 | <0.0005 | 0.92 | 0.88 | 0.96 | <0.0005 | 0.86 | 0.79 | 0.94 | 0.001 |
|  | MH:OC | 0.73 | 0.49 | 1.10 | 0.139 | 0.89 | 0.68 | 1.17 | 0.417 | 0.89 | 0.80 | 1.00 | 0.051 | 0.96 | 0.90 | 1.04 | 0.304 | 0.99 | 0.82 | 1.18 | 0.870 |
|  | Smoker:OC | 0.96 | 0.77 | 1.20 | 0.705 | 1.03 | 0.87 | 1.21 | 0.770 | 0.88 | 0.83 | 0.94 | <0.0005 | 0.93 | 0.89 | 0.97 | <0.0005 | 1.10 | 1.00 | 1.21 | 0.049 |

**Appendix S9– Interventional Disparity Measures**

Table E: Interventional Disparity Measures (IDM) as an absolute percentage change for preterm birth, as a percentage of the total.

|  | Absolute change | Percentage of total |
| --- | --- | --- |
| TAA | 1.3 (-0.4, 2.9) | 21.6 |
| IDM DE | 0.7 (-0.9, 2.2) | 12.3 |
| IDM IE through smoking | 0.3 (0.0, 0.7) | 5.6 |
| IDM IE through maternal mental health | 0.0 (-0.1, 0.1) | 0.3 |
| IDM IE through maternal physical health | 0.1 (-0.0, 0.2) | 1.4 |
| IDM IE through obstetric conditions | 0.1 (-0.0, 0.3) | 1.8 |
| IDM MD | 0.0 (-0.1, 0.1) | 0.1 |

TAA: total adjusted association, DE: direct effect, IE: indirect effect, MD: mediated dependence.

# Appendix S10 – Full regression outputs for controlled direct effect estimates and interventional disparity measures for sensitivity analyses.

Table F: Model results and 95% confidence intervals for total effects logistic non-mediator adjusted regressions (exposure and confounders), for preterm birth (<37 weeks), complete case analysis

|  |  | AOR | LCL | UCL | p-value |
| --- | --- | --- | --- | --- | --- |
| Socioeconomic Status | 1 | 1.20 | 1.14 | 1.26 | <0.0005 |
|  | 2 | 1.11 | 1.05 | 1.16 | <0.0005 |
|  | 3 | 1.01 | 0.96 | 1.06 | 0.655 |
|  | 4 | 0.97 | 0.92 | 1.03 | 0.305 |
|  | 5 | REF | . | . | . |
| Maternal Age | <18 | 1.08 | 0.98 | 1.18 | 0.116 |
|  | 18-24 | REF | . | . | . |
|  | 25-34 | 1.03 | 0.99 | 1.07 | 0.125 |
|  | 35+ | 1.18 | 1.13 | 1.24 | <0.0005 |
| Marital Status | married/civil partnership | REF | . | . | . |
|  | Not married, sole registration | 1.44 | 1.36 | 1.52 | <0.0005 |
|  | Not married, joint registration, parents same address | 1.11 | 1.07 | 1.15 | <0.0005 |
|  | Not married, joint registration, parents different address | 1.33 | 1.27 | 1.40 | <0.0005 |
| Parity | 0 | REF | . | . | . |
|  | 1 | 0.74 | 0.72 | 0.77 | <0.0005 |
|  | 2+ | 1.05 | 1.01 | 1.09 | 0.008 |

Table G: Model results and 95% confidence intervals for total effects logistic mediator adjusted regressions (exposure, mediators and confounders), for preterm birth (<37 weeks), complete case analysis.

|  |  | AOR | LCL | UCL | p-value |
| --- | --- | --- | --- | --- | --- |
| Socioeconomic Status | 1 | 1.14 | 1.07 | 1.23 | <0.0005 |
|  | 2 | 1.09 | 1.01 | 1.16 | 0.019 |
|  | 3 | 0.99 | 0.92 | 1.06 | 0.733 |
|  | 4 | 0.97 | 0.90 | 1.04 | 0.419 |
|  | 5 | REF | . | . | . |
| Smoking During Pregnancy | No | REF | . | . | . |
|  | Yes | 1.43 | 1.27 | 1.60 | <0.0005 |
| Maternal Mental Health | No | REF | . | . | . |
|  | Yes | 1.64 | 1.34 | 1.98 | <0.0005 |
| Maternal Physical Health | No | REF | . | . | . |
|  | Yes | 1.87 | 1.67 | 2.10 | <0.0005 |
| Obstetric Conditions | No | REF | . | . | . |
|  | Yes | 2.21 | 2.03 | 2.40 | <0.0005 |
| Maternal Age | <18 | 1.08 | 0.98 | 1.18 | 0.105 |
|  | 18-24 | REF | . | . | . |
|  | 25-34 | 1.10 | 1.06 | 1.14 | <0.0005 |
|  | 35+ | 1.27 | 1.21 | 1.33 | <0.0005 |
| Marital Status | married/civil partnership | REF | . | . | . |
|  | Not married, sole registration | 1.20 | 1.14 | 1.27 | <0.0005 |
|  | Not married, joint registration, parents same address | 1.04 | 1.00 | 1.07 | 0.038 |
|  | Not married, joint registration, parents different address | 1.17 | 1.11 | 1.22 | <0.0005 |
| Parity | 0 | REF | . | . | . |
|  | 1 | 0.74 | 0.71 | 0.77 | <0.0005 |
|  | 2+ | 0.98 | 0.95 | 1.02 | 0.370 |
| Interaction terms | WIMD 1:Smoker | 1.12 | 0.99 | 1.27 | 0.065 |
|  | WIMD 2:Smoker | 1.03 | 0.90 | 1.17 | 0.683 |
|  | WIMD 3:Smoker | 1.12 | 0.98 | 1.28 | 0.098 |
|  | WIMD 4:Smoker | 1.03 | 0.89 | 1.18 | 0.717 |
|  | WIMD 1:MH | 0.95 | 0.77 | 1.18 | 0.625 |
|  | WIMD 2:MH | 0.90 | 0.72 | 1.13 | 0.356 |
|  | WIMD 3:MH | 0.83 | 0.66 | 1.05 | 0.121 |
|  | WIMD 4:MH | 0.99 | 0.78 | 1.27 | 0.958 |
|  | WIMD 1:PH | 0.86 | 0.75 | 0.98 | 0.020 |
|  | WIMD 2:PH | 0.95 | 0.83 | 1.09 | 0.465 |
|  | WIMD 3:PH | 0.97 | 0.84 | 1.11 | 0.651 |
|  | WIMD 4:PH | 1.04 | 0.90 | 1.20 | 0.631 |
|  | WIMD 1:OC | 0.89 | 0.80 | 0.99 | 0.028 |
|  | WIMD 2:OC | 0.95 | 0.86 | 1.06 | 0.358 |
|  | WIMD 3:OC | 0.98 | 0.88 | 1.09 | 0.654 |
|  | WIMD 4:OC | 0.97 | 0.87 | 1.09 | 0.623 |
|  | Smoker:MH | 0.94 | 0.83 | 1.06 | 0.338 |
|  | PH:MH | 0.93 | 0.81 | 1.08 | 0.356 |
|  | Smoker:PH | 0.94 | 0.86 | 1.02 | 0.134 |
|  | PH:OC | 0.83 | 0.77 | 0.90 | <0.0005 |
|  | MH:OC | 0.88 | 0.78 | 1.00 | 0.052 |
|  | Smoker:OC | 0.87 | 0.82 | 0.94 | <0.0005 |

MH – Maternal Mental Health, PH – Maternal Physical Health, OC – Obstetric Conditions

Table H: Interventional Disparity Measures (IDM) as an absolute percentage change for preterm birth.

|  | Absolute change |
| --- | --- |
| TAA | 1.0 |
| IDM DE | 0.4 |
| IDM IE through smoking | 0.4 |
| IDM IE through maternal mental health | 0.0 |
| IDM IE through maternal physical health | 0.1 |
| IDM IE through obstetric conditions | 0.1 |
| IDM MD | 0.0 |

TAA: total adjusted association, DE: direct effect, IE: indirect effect, MD: mediated dependence, complete case analysis. MH – Maternal Mental Health, PH – Maternal Physical Health, OC – Obstetric Conditions

Table I: Model results and 95% confidence intervals for total effects logistic non-mediator adjusted regressions (exposure and confounders), for preterm birth (<34 weeks)

|  |  | AOR | LCL | UCL | p-value |
| --- | --- | --- | --- | --- | --- |
| Socioeconomic Status | 1 | 1.33 | 1.24 | 1.42 | <0.0005 |
|  | 2 | 1.21 | 1.13 | 1.30 | <0.0005 |
|  | 3 | 1.12 | 1.04 | 1.20 | 0.002 |
|  | 4 | 1.10 | 1.02 | 1.18 | 0.010 |
|  | 5 | REF | . | . | . |
| Maternal Age | <18 | 1.18 | 1.05 | 1.33 | 0.004 |
|  | 18-24 | REF | . | . | . |
|  | 25-34 | 1.09 | 1.03 | 1.14 | 0.001 |
|  | 35+ | 1.27 | 1.19 | 1.36 | <0.0005 |
| Marital Status | married/civil partnership | REF | . | . | . |
|  | Not married, sole registration | 1.47 | 1.36 | 1.59 | <0.0005 |
|  | Not married, joint registration, parents same address | 1.16 | 1.11 | 1.22 | <0.0005 |
|  | Not married, joint registration, parents different address | 1.49 | 1.40 | 1.59 | <0.0005 |
| Parity | 0 | REF | . | . | . |
|  | 1 | 0.63 | 0.60 | 0.66 | <0.0005 |
|  | 2+ | 0.94 | 0.89 | 1.00 | 0.049 |

Table J: Model results and 95% confidence intervals for total effects logistic mediator adjusted regressions (exposure, mediators and confounders), for preterm birth (<34 weeks), complete case analysis.

|  |  | AOR | LCL | UCL | p-value |
| --- | --- | --- | --- | --- | --- |
| Socioeconomic Status | 1 | 1.29 | 1.17 | 1.43 | <0.0005 |
|  | 2 | 1.14 | 1.03 | 1.27 | 0.011 |
|  | 3 | 1.09 | 0.98 | 1.21 | 0.121 |
|  | 4 | 1.11 | 1.00 | 1.23 | 0.057 |
|  | 5 | REF | . | . | . |
| Smoking During Pregnancy | No | REF | . | . | . |
|  | Yes | 1.59 | 1.31 | 1.94 | <0.0005 |
| Maternal Mental Health | No | REF | . | . | . |
|  | Yes | 1.81 | 1.38 | 2.37 | <0.0005 |
| Maternal Physical Health | No | REF | . | . | . |
|  | Yes | 1.78 | 1.52 | 2.09 | <0.0005 |
| Obstetric Conditions | No | REF | . | . | . |
|  | Yes | 2.75 | 2.45 | 3.08 | <0.0005 |
| Maternal Age | <18 | 1.19 | 1.06 | 1.33 | 0.004 |
|  | 18-24 | REF | . | . | . |
|  | 25-34 | 1.17 | 1.11 | 1.23 | <0.0005 |
|  | 35+ | 1.38 | 1.29 | 1.47 | <0.0005 |
| Marital Status | married/civil partnership | REF | . | . | . |
|  | Not married, sole registration | 1.19 | 1.10 | 1.29 | <0.0005 |
|  | Not married, joint registration, parents same address | 1.07 | 1.02 | 1.12 | 0.007 |
|  | Not married, joint registration, parents different address | 1.27 | 1.19 | 1.35 | <0.0005 |
| Parity | 0 | REF | . | . | . |
|  | 1 | 0.63 | 0.60 | 0.66 | <0.0005 |
|  | 2+ | 0.87 | 0.81 | 0.92 | <0.0005 |
| Interaction terms | WIMD 1:Smoker | 1.06 | 0.87 | 1.30 | 0.557 |
|  | WIMD 2:Smoker | 1.10 | 0.89 | 1.36 | 0.372 |
|  | WIMD 3:Smoker | 1.04 | 0.84 | 1.30 | 0.700 |
|  | WIMD 4:Smoker | 0.97 | 0.77 | 1.23 | 0.794 |
|  | WIMD 1:MH | 0.81 | 0.60 | 1.09 | 0.158 |
|  | WIMD 2:MH | 0.84 | 0.62 | 1.14 | 0.255 |
|  | WIMD 3:MH | 0.89 | 0.64 | 1.22 | 0.466 |
|  | WIMD 4:MH | 0.88 | 0.63 | 1.24 | 0.476 |
|  | WIMD 1:PH | 0.89 | 0.74 | 1.05 | 0.166 |
|  | WIMD 2:PH | 1.08 | 0.91 | 1.30 | 0.369 |
|  | WIMD 3:PH | 1.10 | 0.91 | 1.32 | 0.316 |
|  | WIMD 4:PH | 1.10 | 0.91 | 1.34 | 0.310 |
|  | WIMD 1:OC | 0.89 | 0.77 | 1.02 | 0.087 |
|  | WIMD 2:OC | 0.91 | 0.79 | 1.04 | 0.174 |
|  | WIMD 3:OC | 0.94 | 0.81 | 1.09 | 0.409 |
|  | WIMD 4:OC | 0.96 | 0.83 | 1.12 | 0.612 |
|  | Smoker:MH | 0.96 | 0.80 | 1.16 | 0.668 |
|  | PH:MH | 1.01 | 0.83 | 1.23 | 0.915 |
|  | Smoker:PH | 0.90 | 0.80 | 1.02 | 0.103 |
|  | PH:OC | 0.72 | 0.65 | 0.80 | <0.0005 |
|  | MH:OC | 0.80 | 0.68 | 0.95 | 0.012 |
|  | Smoker:OC | 0.92 | 0.82 | 1.02 | 0.112 |

MH – Maternal Mental Health, PH – Maternal Physical Health, OC – Obstetric Conditions

Table K: Interventional Disparity Measures (IDM) as an absolute percentage change for preterm birth, as a percentage of the total.

|  | Absolute change | Percentage of total |
| --- | --- | --- |
| TAA | 0.5 | 27.0 |
| IDM DE | 0.3 | 15.0 |
| IDM IE through smoking | 0.1 | 7.5 |
| IDM IE through maternal mental health | 0.0 | 0.3 |
| IDM IE through maternal physical health | 0.0 | 1.6 |
| IDM IE through obstetric conditions | 0.0 | 2.5 |
| IDM MD | 0.0 | 0.1 |

TAA: total adjusted association, DE: direct effect, IE: indirect effect, MD: mediated dependence

Table L: Panel A - Proportion Eliminated and Proportion Mediated estimates for socioeconomic inequalities in Preterm Birth for complete case analysis. Proportion eliminated calculated through Controlled Direct Effects, including all mediators, and Proportion Mediated calculated through Interventional Disparity Measures. Panel B – imputed analysis using 34 weeks as the cut off for Preterm Birth.

| A | |
| --- | --- |
| Proportion Eliminated – of the effect of low SES (WIMD quintile 1 vs 5) through the removal of mediators | 27.0 (-2.7 to 59.0) |
| Proportion of low SES (WIMD quintile 1 vs 5) association Mediated by Smoking | 37.7 |
| Proportion of low SES (WIMD quintile 1 vs 5) association Mediated by Maternal Mental Ill Health | 3.0 |
| Proportion of low SES (WIMD quintile 1 vs 5) association Mediated by Maternal Physical Health Issues | 8.5 |
| Proportion of low SES (WIMD quintile 1 vs 5) association Mediated by Maternal Obstetric Conditions | 10.4 |
| B | |
| Proportion Eliminated – of the effect of low SES (WIMD quintile 1 vs 5) through the removal of mediators | 12.2 |
| Proportion of low SES (WIMD quintile 1 vs 5) association Mediated by Smoking | 27.8 |
| Proportion of low SES (WIMD quintile 1 vs 5) association Mediated by Maternal Mental Ill Health | 1.2 |
| Proportion of low SES (WIMD quintile 1 vs 5) association Mediated by Maternal Physical Health Issues | 5.8 |
| Proportion of low SES (WIMD quintile 1 vs 5) association Mediated by Maternal Obstetric Conditions | 9.2 |

Table M: Model results and 95% confidence intervals for total effects logistic non-mediator adjusted regressions (exposure and confounders), for preterm birth (<37 weeks), with year included.

|  |  | AOR | LCL | UCL | p-value |
| --- | --- | --- | --- | --- | --- |
| Socioeconomic Status | 1 | 1.27 | 1.22 | 1.31 | <0.0005 |
|  | 2 | 1.18 | 1.14 | 1.22 | <0.0005 |
|  | 3 | 1.11 | 1.07 | 1.15 | <0.0005 |
|  | 4 | 1.06 | 1.02 | 1.10 | 0.005 |
|  | 5 | REF | . | . | . |
| Maternal Age | <18 | 1.12 | 1.05 | 1.20 | 0.001 |
|  | 18-24 | REF | . | . | . |
|  | 25-34 | 1.04 | 1.01 | 1.07 | 0.003 |
|  | 35+ | 1.22 | 1.17 | 1.26 | <0.0005 |
| Marital Status | married/civil partnership | REF | . | . | . |
|  | Not married, sole registration | 1.41 | 1.36 | 1.47 | <0.0005 |
|  | Not married, joint registration, parents same address | 1.12 | 1.09 | 1.15 | <0.0005 |
|  | Not married, joint registration, parents different address | 1.32 | 1.27 | 1.36 | <0.0005 |
| Parity | 0 | REF | . | . | . |
|  | 1 | 0.74 | 0.72 | 0.77 | <0.0005 |
|  | 2+ | 1.03 | 1.00 | 1.06 | 0.045 |
| Year | 2000 | REF | . | . | . |
|  | 2001 | 0.98 | 0.91 | 1.06 | 0.688 |
|  | 2002 | 1.07 | 0.99 | 1.15 | 0.079 |
|  | 2003 | 1.10 | 1.03 | 1.18 | 0.007 |
|  | 2004 | 1.07 | 1.00 | 1.15 | 0.066 |
|  | 2005 | 0.97 | 0.90 | 1.04 | 0.411 |
|  | 2006 | 0.99 | 0.92 | 1.06 | 0.694 |
|  | 2007 | 1.00 | 0.94 | 1.08 | 0.914 |
|  | 2008 | 0.94 | 0.88 | 1.01 | 0.076 |
|  | 2009 | 0.96 | 0.89 | 1.03 | 0.238 |
|  | 2010 | 0.92 | 0.86 | 0.99 | 0.018 |
|  | 2011 | 0.94 | 0.88 | 1.01 | 0.071 |
|  | 2012 | 0.90 | 0.84 | 0.96 | 0.002 |
|  | 2013 | 0.90 | 0.84 | 0.97 | 0.005 |
|  | 2014 | 0.92 | 0.86 | 0.99 | 0.021 |
|  | 2015 | 0.92 | 0.86 | 0.99 | 0.029 |
|  | 2016 | 0.99 | 0.93 | 1.07 | 0.879 |
|  | 2017 | 1.04 | 0.97 | 1.12 | 0.254 |
|  | 2018 | 1.05 | 0.98 | 1.12 | 0.186 |
|  | 2019 | 1.08 | 1.00 | 1.16 | 0.056 |

Table N: Model results and 95% confidence intervals for total effects logistic mediator adjusted regressions (exposure, mediators and confounders), for preterm birth (<37 weeks) with year included.

|  |  | AOR | LCL | UCL | p-value |
| --- | --- | --- | --- | --- | --- |
| Socioeconomic Status | 1 | 1.21 | 1.15 | 1.28 | <0.0005 |
|  | 2 | 1.14 | 1.08 | 1.20 | <0.0005 |
|  | 3 | 1.08 | 1.02 | 1.14 | 0.005 |
|  | 4 | 1.05 | 1.00 | 1.11 | 0.074 |
|  | 5 | REF | . | . | . |
| Smoking During Pregnancy | No | REF | . | . | . |
|  | Yes | 1.45 | 1.30 | 1.61 | <0.0005 |
| Maternal Mental Health | No | REF | . | . | . |
|  | Yes | 1.54 | 1.31 | 1.80 | <0.0005 |
| Maternal Physical Health | No | REF | . | . | . |
|  | Yes | 1.76 | 1.62 | 1.92 | <0.0005 |
| Obstetric Conditions | No | REF | . | . | . |
|  | Yes | 2.14 | 2.01 | 2.28 | <0.0005 |
| Maternal Age | <18 | 1.12 | 1.05 | 1.20 | 0.001 |
|  | 18-24 | REF | . | . | . |
|  | 25-34 | 1.11 | 1.08 | 1.14 | <0.0005 |
|  | 35+ | 1.31 | 1.26 | 1.36 | <0.0005 |
| Marital Status | married/civil partnership | REF | . | . | . |
|  | Not married, sole registration | 1.19 | 1.14 | 1.24 | <0.0005 |
|  | Not married, joint registration, parents same address | 1.05 | 1.02 | 1.08 | <0.0005 |
|  | Not married, joint registration, parents different address | 1.16 | 1.11 | 1.20 | <0.0005 |
| Parity | 0 | REF | . | . | . |
|  | 1 | 0.74 | 0.72 | 0.76 | <0.0005 |
|  | 2+ | 0.97 | 0.93 | 1.00 | 0.033 |
| Year | 2000 | REF | . | . | . |
|  | 2001 | 0.97 | 0.90 | 1.05 | 0.477 |
|  | 2002 | 1.07 | 0.99 | 1.15 | 0.082 |
|  | 2003 | 1.12 | 1.04 | 1.20 | 0.002 |
|  | 2004 | 1.08 | 1.01 | 1.16 | 0.031 |
|  | 2005 | 1.00 | 0.93 | 1.07 | 0.951 |
|  | 2006 | 1.02 | 0.95 | 1.09 | 0.583 |
|  | 2007 | 1.04 | 0.97 | 1.12 | 0.222 |
|  | 2008 | 0.97 | 0.90 | 1.04 | 0.374 |
|  | 2009 | 0.97 | 0.91 | 1.04 | 0.411 |
|  | 2010 | 0.93 | 0.87 | 1.00 | 0.043 |
|  | 2011 | 0.94 | 0.88 | 1.01 | 0.095 |
|  | 2012 | 0.89 | 0.83 | 0.96 | 0.002 |
|  | 2013 | 0.90 | 0.84 | 0.97 | 0.003 |
|  | 2014 | 0.92 | 0.85 | 0.99 | 0.018 |
|  | 2015 | 0.92 | 0.86 | 0.99 | 0.024 |
|  | 2016 | 0.99 | 0.92 | 1.06 | 0.777 |
|  | 2017 | 1.03 | 0.96 | 1.11 | 0.386 |
|  | 2018 | 1.03 | 0.96 | 1.11 | 0.389 |
|  | 2019 | 1.02 | 0.94 | 1.10 | 0.606 |
| Interaction terms | WIMD 1:Smoker | 1.06 | 0.94 | 1.20 | 0.350 |
|  | WIMD 2:Smoker | 1.06 | 0.95 | 1.19 | 0.282 |
|  | WIMD 3:Smoker | 1.08 | 0.95 | 1.23 | 0.217 |
|  | WIMD 4:Smoker | 1.03 | 0.91 | 1.18 | 0.617 |
|  | WIMD 1:MH | 0.95 | 0.80 | 1.13 | 0.567 |
|  | WIMD 2:MH | 0.89 | 0.75 | 1.07 | 0.211 |
|  | WIMD 3:MH | 0.85 | 0.70 | 1.02 | 0.085 |
|  | WIMD 4:MH | 1.04 | 0.86 | 1.27 | 0.686 |
|  | WIMD 1:PH | 0.89 | 0.81 | 0.98 | 0.020 |
|  | WIMD 2:PH | 0.98 | 0.89 | 1.08 | 0.666 |
|  | WIMD 3:PH | 1.01 | 0.91 | 1.11 | 0.917 |
|  | WIMD 4:PH | 1.05 | 0.94 | 1.16 | 0.414 |
|  | WIMD 1:OC | 0.93 | 0.86 | 1.00 | 0.061 |
|  | WIMD 2:OC | 0.94 | 0.87 | 1.02 | 0.117 |
|  | WIMD 3:OC | 0.97 | 0.90 | 1.05 | 0.501 |
|  | WIMD 4:OC | 0.97 | 0.89 | 1.06 | 0.508 |
|  | Smoker:MH | 0.97 | 0.87 | 1.09 | 0.601 |
|  | PH:MH | 0.98 | 0.87 | 1.09 | 0.667 |
|  | Smoker:PH | 0.92 | 0.85 | 0.99 | 0.019 |
|  | PH:OC | 0.86 | 0.81 | 0.91 | <0.0005 |
|  | MH:OC | 0.88 | 0.79 | 0.97 | 0.012 |
|  | Smoker:OC | 0.91 | 0.85 | 0.97 | 0.002 |

MH – Maternal Mental Health, PH – Maternal Physical Health, OC – Obstetric Conditions

Table O: Model results and 95% confidence intervals for total effects logistic non-mediator adjusted regressions (exposure and confounders), for preterm birth (<37 weeks), with marital status not included.

|  |  | AOR | LCL | UCL | p-value |
| --- | --- | --- | --- | --- | --- |
| Socioeconomic Status | 1 | 1.34 | 1.29 | 1.38 | 0.000 |
|  | 2 | 1.22 | 1.18 | 1.27 | 0.000 |
|  | 3 | 1.13 | 1.09 | 1.17 | 0.000 |
|  | 4 | 1.07 | 1.03 | 1.11 | 0.001 |
|  | 5 | REF | . | . | . |
| Maternal Age | <18 | 1.21 | 1.14 | 1.30 | 0.000 |
|  | 18-24 | REF | . | . | . |
|  | 25-34 | 0.96 | 0.93 | 0.98 | 0.002 |
|  | 35+ | 1.11 | 1.07 | 1.15 | 0.000 |
| Parity | 0 | REF | . | . | . |
|  | 1 | 0.74 | 0.72 | 0.76 | 0.000 |
|  | 2+ | 1.04 | 1.01 | 1.08 | 0.006 |

Table P: Model results and 95% confidence intervals for total effects logistic mediator adjusted regressions (exposure, mediators and confounders), for preterm birth (<37 weeks) with marital status not included.

|  |  | AOR | LCL | UCL | p-value |
| --- | --- | --- | --- | --- | --- |
| Socioeconomic Status | 1 | 1.23 | 1.17 | 1.30 | 0.000 |
|  | 2 | 1.16 | 1.10 | 1.22 | 0.000 |
|  | 3 | 1.09 | 1.03 | 1.15 | 0.002 |
|  | 4 | 1.05 | 1.00 | 1.11 | 0.061 |
|  | 5 |  |  |  |  |
| Smoking During Pregnancy | No |  |  |  |  |
|  | Yes | 1.49 | 1.34 | 1.65 | 0.000 |
| Maternal Mental Health | No |  |  |  |  |
|  | Yes | 1.56 | 1.34 | 1.82 | 0.000 |
| Maternal Physical Health | No |  |  |  |  |
|  | Yes | 1.77 | 1.62 | 1.93 | 0.000 |
| Obstetric Conditions | No |  |  |  |  |
|  | Yes | 2.14 | 2.01 | 2.28 | 0.000 |
| Maternal Age | <18 | 1.18 | 1.10 | 1.26 | 0.000 |
|  | 18-24 |  |  |  |  |
|  | 25-34 | 1.07 | 1.04 | 1.10 | 0.000 |
|  | 35+ | 1.25 | 1.21 | 1.30 | 0.000 |
| Parity | 0 |  |  |  |  |
|  | 1 | 0.74 | 0.72 | 0.76 | 0.000 |
|  | 2+ | 0.97 | 0.94 | 1.00 | 0.069 |
| Interaction terms | WIMD 1:Smoker | 1.06 | 0.94 | 1.20 | 0.323 |
|  | WIMD 2:Smoker | 1.06 | 0.95 | 1.19 | 0.268 |
|  | WIMD 3:Smoker | 1.09 | 0.96 | 1.23 | 0.205 |
|  | WIMD 4:Smoker | 1.03 | 0.91 | 1.18 | 0.621 |
|  | WIMD 1:MH | 0.95 | 0.80 | 1.13 | 0.576 |
|  | WIMD 2:MH | 0.89 | 0.75 | 1.07 | 0.208 |
|  | WIMD 3:MH | 0.84 | 0.70 | 1.02 | 0.079 |
|  | WIMD 4:MH | 1.04 | 0.86 | 1.27 | 0.684 |
|  | WIMD 1:PH | 0.89 | 0.81 | 0.98 | 0.020 |
|  | WIMD 2:PH | 0.98 | 0.89 | 1.08 | 0.684 |
|  | WIMD 3:PH | 1.01 | 0.91 | 1.11 | 0.915 |
|  | WIMD 4:PH | 1.04 | 0.94 | 1.16 | 0.420 |
|  | WIMD 1:OC | 0.93 | 0.86 | 1.00 | 0.060 |
|  | WIMD 2:OC | 0.94 | 0.87 | 1.02 | 0.117 |
|  | WIMD 3:OC | 0.97 | 0.90 | 1.05 | 0.494 |
|  | WIMD 4:OC | 0.97 | 0.89 | 1.06 | 0.514 |
|  | Smoker:MH | 0.97 | 0.86 | 1.08 | 0.537 |
|  | PH:MH | 0.98 | 0.87 | 1.10 | 0.687 |
|  | Smoker:PH | 0.92 | 0.85 | 0.98 | 0.016 |
|  | PH:OC | 0.86 | 0.81 | 0.91 | 0.000 |
|  | MH:OC | 0.88 | 0.80 | 0.97 | 0.012 |
|  | Smoker:OC | 0.91 | 0.85 | 0.96 | 0.002 |

MH – Maternal Mental Health, PH – Maternal Physical Health, OC – Obstetric Conditions

Table Q: Model results and 95% confidence intervals for total effects logistic mediator adjusted regressions (exposure, mediators and confounders), for preterm birth (<37 weeks) with smoking split into three categories.

|  |  | AOR | LCL | UCL | p-value |
| --- | --- | --- | --- | --- | --- |
| Socioeconomic Status | 1 | 1.21 | 1.14 | 1.28 | 0.000 |
|  | 2 | 1.13 | 1.07 | 1.20 | 0.000 |
|  | 3 | 1.07 | 1.01 | 1.13 | 0.024 |
|  | 4 | 1.04 | 0.98 | 1.10 | 0.171 |
|  | 5 | REF | . | . | . |
| Smoking During Pregnancy | No | REF | . | . | . |
|  | Current | 1.44 | 1.29 | 1.61 | 0.000 |
|  | Ex-smoker | 0.96 | 0.86 | 1.06 | 0.422 |
| Maternal Mental Health | No | REF | . | . | . |
|  | Yes | 1.53 | 1.30 | 1.80 | 0.000 |
| Maternal Physical Health | No | REF | . | . | . |
|  | Yes | 1.78 | 1.63 | 1.95 | 0.000 |
| Obstetric Conditions | No | REF | . | . | . |
|  | Yes | 2.15 | 2.01 | 2.29 | 0.000 |
| Maternal Age | <18 | 1.13 | 1.06 | 1.21 | 0.000 |
|  | 18-24 | REF | . | . | . |
|  | 25-34 | 1.11 | 1.08 | 1.14 | 0.000 |
|  | 35+ | 1.30 | 1.25 | 1.35 | 0.000 |
| Marital Status | married/civil partnership | REF | . | . | . |
|  | Not married, sole registration | 1.19 | 1.14 | 1.25 | 0.000 |
|  | Not married, joint registration, parents same address | 1.04 | 1.02 | 1.07 | 0.002 |
|  | Not married, joint registration, parents different address | 1.15 | 1.11 | 1.19 | 0.000 |
| Parity | 0 | REF | . | . | . |
|  | 1 | 0.74 | 0.72 | 0.77 | 0.000 |
|  | 2+ | 0.97 | 0.94 | 1.00 | 0.075 |
| Interaction terms | WIMD 1:Current Smoker | 1.05 | 0.93 | 1.18 | 0.416 |
|  | WIMD 2:Current Smoker | 1.06 | 0.94 | 1.19 | 0.350 |
|  | WIMD 3:Current Smoker | 1.08 | 0.95 | 1.23 | 0.253 |
|  | WIMD 4:Current Smoker | 1.03 | 0.89 | 1.20 | 0.638 |
|  | WIMD 1:Ex-Smoker | 1.02 | 0.90 | 1.17 | 0.716 |
|  | WIMD 2:Ex-Smoker | 1.08 | 0.95 | 1.23 | 0.237 |
|  | WIMD 3:Ex-Smoker | 1.09 | 0.95 | 1.25 | 0.236 |
|  | WIMD 4:Ex-Smoker | 1.05 | 0.92 | 1.20 | 0.457 |
|  | WIMD 1:MH | 0.95 | 0.80 | 1.12 | 0.549 |
|  | WIMD 2:MH | 0.89 | 0.74 | 1.06 | 0.191 |
|  | WIMD 3:MH | 0.84 | 0.70 | 1.02 | 0.073 |
|  | WIMD 4:MH | 1.04 | 0.85 | 1.26 | 0.712 |
|  | WIMD 1:PH | 0.89 | 0.81 | 0.98 | 0.018 |
|  | WIMD 2:PH | 0.98 | 0.88 | 1.08 | 0.647 |
|  | WIMD 3:PH | 1.00 | 0.90 | 1.11 | 0.972 |
|  | WIMD 4:PH | 1.04 | 0.94 | 1.16 | 0.447 |
|  | WIMD 1:OC | 0.93 | 0.86 | 1.00 | 0.056 |
|  | WIMD 2:OC | 0.94 | 0.87 | 1.02 | 0.115 |
|  | WIMD 3:OC | 0.97 | 0.90 | 1.05 | 0.479 |
|  | WIMD 4:OC | 0.97 | 0.89 | 1.06 | 0.507 |
|  | Current Smoker:MH | 0.98 | 0.88 | 1.10 | 0.727 |
|  | Ex-Smoker:MH | 1.01 | 0.86 | 1.19 | 0.883 |
|  | PH:MH | 0.98 | 0.87 | 1.10 | 0.710 |
|  | Current Smoker:PH | 0.91 | 0.85 | 0.98 | 0.011 |
|  | Ex-Smoker:PH | 0.93 | 0.84 | 1.03 | 0.186 |
|  | PH:OC | 0.86 | 0.81 | 0.91 | 0.000 |
|  | MH:OC | 0.88 | 0.80 | 0.97 | 0.014 |
|  | Current Smoker:OC | 0.91 | 0.86 | 0.96 | 0.001 |
|  | Ex-Smoker:OC | 0.97 | 0.89 | 1.06 | 0.563 |

MH – Maternal Mental Health, PH – Maternal Physical Health, OC – Obstetric Conditions
